# The Health Care Sector’s Experience of Blockchain: A Cross-disciplinary Investigation of Its Real Transformative Potential

**DOI:** 10.2196/24109

**Published:** 2021-12-20

**Authors:** Karen Yeung

**Affiliations:** 1 Birmingham Law School and School of Computer Science University of Birmingham Birmingham United Kingdom

**Keywords:** blockchain, health information management, health information systems, electronic health record, data sharing, health services administration, privacy of patient data, computer security, mobile phone

## Abstract

**Background:**

Academic literature highlights blockchain’s potential to transform health care, particularly by seamlessly and securely integrating existing *data silos* while enabling patients to exercise automated, fine-grained control over access to their electronic health records. However, no serious scholarly attempt has been made to assess how these technologies have *in fact* been applied to real-world health care contexts.

**Objective:**

The primary aim of this paper is to assess whether blockchain’s theoretical potential to deliver transformative benefits to health care is likely to become a reality by undertaking a critical investigation of the health care sector’s actual experience of blockchain technologies to date.

**Methods:**

This mixed methods study entailed a series of iterative, in-depth, theoretically oriented, desk-based investigations and 2 focus group investigations. It builds on the findings of a companion research study documenting real-world engagement with blockchain technologies in health care. Data were sourced from academic and gray literature from multiple disciplinary perspectives concerned with the configuration, design, and functionality of blockchain technologies. The analysis proceeded in 3 stages. First, it undertook a qualitative investigation of observed patterns of blockchain for health care engagement to identify the application domains, data-sharing problems, and the challenges encountered to date. Second, it critically compared these experiences with claims about blockchain’s potential benefits in health care. Third, it developed a theoretical account of challenges that arise in implementing blockchain in health care contexts, thus providing a firmer foundation for appraising its future prospects in health care.

**Results:**

Health care organizations have actively experimented with blockchain technologies since 2016 and have demonstrated proof of concept for several applications (*use cases*) primarily concerned with administrative data and to facilitate medical research by enabling algorithmic models to be trained on multiple disparately located sets of patient data in a secure, privacy-preserving manner. However, blockchain technology is yet to be implemented at scale in health care, remaining largely in its infancy. These early experiences have demonstrated blockchain’s potential to generate meaningful value to health care by facilitating data sharing between organizations in circumstances where computational trust can overcome a lack of social trust that might otherwise prevent valuable cooperation. Although there are genuine prospects of using blockchain to bring about positive transformations in health care, the successful development of blockchain for health care applications faces a number of very significant, multidimensional, and highly complex challenges. Early experience suggests that blockchain is unlikely to rapidly and radically revolutionize health care.

**Conclusions:**

The successful development of blockchain for health care applications faces numerous significant, multidimensional, and complex challenges that will not be easily overcome, suggesting that blockchain technologies are unlikely to revolutionize health care in the near future.

## Introduction

### Background

In recent years, distributed ledger technologies commonly referred to as *blockchain* have generated considerable interest and excitement across many industries, including health care, supported by claims of their radically disruptive and transformative potential. Academic literature focusing on blockchain’s potential to solve health care problems has proliferated [1-7]. Although scholars have identified many possible applications of blockchain in health care [8], blockchain’s perceived value ultimately rests on its potential to create a highly secure, tamper-proof, auditable electronic ledger that can enable responsive, fine-grained, and privacy-respecting access to and sharing of health care data. Accordingly, blockchain is often portrayed as a technological solution that can overcome existing barriers to health information exchange [8], one of the most stubborn challenges that continues to plague contemporary health care [9]. Blockchain advocates claim that these technologies will generate higher quality, more trustworthy, and readily accessible data, which can drive improvements across health care [10]. These improvements would then lead to (1) better quality medical care, including improvements in clinical decision-making and more effective public health management and disease prevention [11]; (2) more efficient, cost-effective, and timely health care administration; and (3) improvements in medical and health care research, resulting from more accurate and secure clinical trial data management and storage [12]. In particular, many believe blockchain will enable fine-grained, patient-controlled access, sharing, and management of electronic health records (EHRs), thereby overcoming existing problems associated with the current *siloed* approach to the storage and management of patient data, which is often regarded as blockchain’s *favorite use case* for health care [13,14].

### The Blockchain for Health Care Promise: Rhetoric or Reality?

However, expecting blockchain technologies to provide an effective, efficient, patient-centered solution to the multiplicity of problems associated with health care data management and sharing is a tall order. This was one of the expected benefits of EHRs; however, despite their widespread take-up in health care settings, ethically sensitive, lawful, timely, and secure sharing and management of health care data remain a critical but seemingly intractable challenge [9,15,16]. Academic literature concerned with health care applications remains overwhelmingly centered on blockchain’s *potential,* focusing on identifying the possible range of benefits that *could* be delivered. However, no serious scholarly attempt has been made to assess the extent to which these technologies have *in fact* been applied to real-world health care contexts and with what consequences, with little attention paid to real-world implementation challenges [17]. In short, academic scholarship remains largely speculative (Multimedia Appendix 1 [1,3,7,18]).

### Technical Dimensions of Blockchain Technologies

#### Overview

To identify what blockchain technologies can and cannot realistically deliver, it is necessary to understand what these technologies are and how they function. Although computer security specialists ascribe a particular technical meaning to *blockchain technologies*, they are understood in a much looser, broader sense for the purposes of this study. In this paper, blockchain technologies refer to a time-stamped database, duplicated across a distributed network of computers (each computer in the network is called a *node*), which is configured such that its technical architecture and operation effectively prevent the rewriting and removal of prior entries. At a basic level, blockchains enable a community of users to record transactions (ie, an interaction between parties) in a shared ledger such that, under the normal operation of the blockchain network, no transaction record can be altered once published. These systems can, as the Bitcoin blockchain demonstrates, enable transactions between strangers without the need for a conventional trusted third-party intermediary, such as a bank or government. Instead, transactions between parties with no pre-existing relationship of trust are enabled by 4 key characteristics of blockchain technologies [19].

#### Ledger

The technology relies on an append-only ledger to provide a complete transactional history, which, because of its technological design, is almost impossible to amend or alter. This differs from a traditional database in which transactions and entries can be altered or overridden.

#### Tamper-Proof

Blockchains use cryptographic methods that rely upon mathematical consensus to confirm the consistency and technical authenticity of each transaction’s digital record, which is then permanently recorded on the ledger and cannot be altered or deleted. The security and accuracy of the ledger are maintained through the use of cryptographic *keys* and signatures that automatically control who can do what with the ledger. If conflicts between different copies of the database arise (eg, because someone is trying to tamper with the data), the automatic consensus mechanism is designed to ensure that only transactions that are consistent with the earlier, stored version of the database are updated and permanently recorded. Distributed ledger systems that take the blockchain form aggregate transactions into *blocks,* and these are added to a *chain* of existing blocks using a cryptographic signature (hence the name *blockchain*). As the data stored on the ledger can be mathematically attested and cannot be tampered with, it is highly secure.

#### Shared

A copy of the ledger is replicated and shared across multiple participating nodes, providing transparency across the node participants in the network.

#### Distributed

As more nodes are added across the network, the ledger becomes increasingly resistant to malicious attacks because it becomes more difficult to interfere with the intended operation of the consensus protocol through which transactions are validated and appended to the ledger. The resilience of the ledger is rooted in its distributed nature. In contrast, conventional databases are based on a centralized, hierarchical design, thus creating a single point of failure.

Blockchain systems can be designed to operate in many ways because of the malleability of the software and protocols through which they are configured. Blockchains may be *permissionless* or *permissioned*, referring to who is entitled to become a network node. Within permissionless blockchain networks, anyone with a computer that has sufficient computing capacity can download the software and participate in the consensus process, storing and updating the shared ledger (referred to as *write* access) without needing permission to do so. As write access is *open* to all for permissionless blockchains, anyone can also *read* or otherwise inspect the ledger. In contrast, permissioned blockchains restrict write access: only those granted advance permission by the relevant authority within the network can participate in the consensus process through which the shared ledger is updated and stored. For permissioned blockchains, the ability of others to *read* and inspect the ledger may be *open* to anyone or can be restricted or *closed* so that only those with authorized access may read its contents. Permissioned blockchains can provide similar functionality to permissionless blockchains but are considerably less computationally demanding. As participating nodes must first be identified and authorized before joining a permissioned network, this provides an assurance of trust across the network, backed by the threat that write access can be withdrawn from misbehaving nodes. As a result, consensus models applied within permissioned blockchains need not provide the same high levels of mathematical and computational security. Therefore, they offer faster performance and are computationally less expensive [19].

The elasticity and malleability of blockchain systems, including the variety of different forms and functionalities that they can offer, has resulted in considerable variation in the way the term *blockchain* is used, particularly in discussions about blockchain for health care. The very broad definition of blockchain adopted here reflects the health care sector use of the term, encompassing any sociotechnical system that uses a distributed, append-only database that relies on cryptographic methods to verify and validate transactions before they are added to the ledger, including both permissioned and permissionless systems, which include distributed ledger technologies that do not aggregate and store data into linked blocks [13].

### Aims, Objectives, and Research Questions

The aim of this study is to critically investigate the health care sector’s engagement with blockchain technologies to date, analyzing those experiences from a theoretical, cross-disciplinary perspective to ground a more realistic appraisal of the potential benefits, difficulties, and implications of implementing blockchain in health care settings. This investigation was animated by the following research questions:

1. To what extent and in what ways have blockchain technologies been taken up in the health care sector?
2. What are the primary real-world opportunities and challenges that blockchain technology generates in health care contexts?
3. What are the prospects of blockchain take-up and implementation in health care settings to solve real-world problems in the short to medium term?

Taken together, these questions can be amalgamated into a single overarching research question: what does health care’s actual experience of blockchain technologies to date reveal about the technologies’ *real* revolutionary potential [20]?

This paper proceeds by outlining the study’s design, data sources, analytic approach, and theoretical foundations. It then sets out its findings by first providing a qualitative overview and explanation of the extent to which blockchain technologies have been taken up in the health care domain. Second, it offers a critical examination of a series of multidimensional challenges, organized thematically, that constitute (or are likely to constitute) very substantial hurdles that must be overcome if blockchain is to be widely taken up in health care settings. Finally, it discusses these challenges in light of the most promising opportunities for generating real value in health care settings and suggests that the use of blockchain to provide fine-grained patient control over medical records is unlikely to live up to its perceived promise.

## Methods

### Study Design and Data Sources

The mixed methods study reported in this paper forms the major component of a Wellcome Trust–funded cross-disciplinary project aimed at understanding the challenges, risks, opportunities, and experiences of the health care sector in using blockchain technologies. It took the form of a theoretical investigation, building upon the initial research study by Motsi-Omoijiade and Kharlamov [21] that sought to identify and map evidence of real-world engagement with blockchain technologies in the health care sector, classifying them according to their context of application (as patient care support, administration, or research data management), level of maturity, and the primary geographic region from which the application appeared to originate. The integrated catalog of health care blockchain applications and use cases created in that study provided a snapshot of the state of health care blockchain take-up on November 30, 2019. It identified 128 *applications*, referring to real-world instances in which blockchain technology has been developed into a commercially viable health-related service created largely from a web-based review of English language websites, gray literature, and academic papers (Multimedia Appendix 2 [21-28]). This study builds on the results of that earlier mapping and classification exercise by drawing on 2 focus group investigations and a series of iterative, in-depth, theoretically oriented desk-based investigations.

### Focus Group Workshops

The 2 focus group discussions were organized in which a mix of both academic researchers and blockchain-for-health care sector experts participated. The first workshop, hosted at the outset of the project, was designed to generate insights that could inform and guide the direction of the study’s inquiries and help understand the current state of health care sector engagement and experience with blockchain technologies for specific health care purposes. The second workshop occurred toward the end of the study. Its aim was to share the provisional study findings with participants to elicit their critical feedback and provoke discussion. The focus group participants were identified and invited with the aim of bringing wide-ranging, relevant expertise to the discussion, given the project’s aim of adopting a multidisciplinary perspective to address the project’s research questions. This approach to recruitment reflects a belief that technical, legal, ethical, management, clinical care, and health service industry knowledge and experience would be needed to successfully apply blockchain to real-world settings.

The participant invitation list was compiled from the principal investigator’s existing knowledge of academic experts with existing or related interests in blockchain from several disciplinary backgrounds, combined with a light touch review of recent academic and digital health care industry publications to identify key health care sector experts with direct and ongoing experience in seeking to apply blockchain technologies to health care. This initial list was supplemented by the use of snowball sampling techniques (asking each academic and industry invitee to nominate 1 or 2 colleagues) to identify others with relevant expertise. The specific composition of the workshops differed in light of their different objectives and participant availability: the initial workshop comprised more academics than industry experts, whereas the final workshop was attended predominantly by industry experts relative to academics (Multimedia Appendix 3).

### Desk-Based Review and Theoretical Investigation

The project’s theoretical, desk-based investigations primarily drew on 2 data sources. First, the investigations drew from a wide variety of academic literature from multiple disciplinary perspectives, including computer science, computer security, digital health, organizational management, operations research, medical law, data protection law, medicine, medical sociology, and innovation studies. Given that relatively little information about health-related blockchain projects is publicized via academic journals [29], and existing scholarly literature on blockchain in health care settings has not hitherto been concerned with real-world engagement and experience, relevant academic sources were identified by deductive critical reflection. This entailed developing and refining a series of thematically focused investigations aimed at acquiring a deeper, more fine-grained, cross-disciplinary understanding of the major issues and challenges that would invariably confront those endeavoring to apply blockchain to health care settings. These included matters of data security and integrity, health information technology (IT) implementation, user resistance, internal and external governance, interoperability, organizational management, and data privacy. Second, gray literature (including observations and reflections from those with direct experience of the health sector’s engagement with blockchain to date) provided a vitally important source of insight, given that the published scholarly literature represents only a small snapshot of global blockchain activity. This literature included reflections collected from various informal sources, such as guidance and opinion pieces published in various formats, including essay collections [30], blog posts, news articles, newsletters by industry bodies (particularly those published by the Healthcare Information Management and Systems Society [HIMSS]) and key industry experts from organizations offering blockchain for health care consultancy services such as Consensys Health and Hashed Health.

### Analytical Approach

This study proceeded from the premise that blockchain’s reliance upon cryptographic techniques and networked, distributed computing facilities to provide a highly secure, shared but tamper-proof database means that its foundational value lies in its capacity to facilitate *secure data sharing and collaboration*, particularly between parties in circumstances where a lack of social trust would otherwise inhibit cooperation. So understood, the development, implementation, and scale up of blockchain technologies into health care contexts are likely to pose distinctive and novel challenges because of both its technological design and the health care contexts in which attempts are being made to implement it.

The analysis proceeded in 3 stages. First, it began by seeking to acquire a deeper, more qualitatively oriented understanding of the observed patterns of blockchain for health care engagement mapped from the Motsi-Omoijiade and Kharlamov [21] study. These first-stage inquiries drew heavily on insights and observations contained in industry publications [29], government reports, mainstream media, and initial focus group discussions. The resulting analysis produced a clearer, richer, and more nuanced understanding of (1) how blockchain technologies were intended to provide specific functional capacities across a variety of real-world health care contexts; (2) the range of applications, context-domains, and participants who were expected to engage with and those expected to benefit from these applications; (3) how blockchain was expected to address specific data-sharing problems encountered in health care settings; and (4) the wide-ranging and multidimensional difficulties or *challenges* that have been encountered in the course of these engagements to date or that can be expected to arise if those engagements continue to expand and deepen, particularly if they extend into clinical settings.

The second-stage analysis entailed a critical comparison of the benefits, difficulties, and drawbacks identified in stage 1 against the claims appearing in the academic literature about blockchain’s potential benefits and shortcomings (both generally and those specific to health care). This analysis sought to evaluate these claims, which were focused almost exclusively on the benefits of blockchain, with little mention or consideration of shortcomings, against early-stage experiences with blockchain implementation in real-world health care settings. The resulting mismatch between academic claims and actual experiences of the technology prompted further scrutinization and critical analysis of the implementation problems identified from the first-stage analysis, grouping them together thematically (rather than primarily in relation to specific functional applications or particular kinds of health care data). This grouping was based on recurring difficulties and challenges that have been encountered (or are likely to arise in relation to specific health care applications, particularly those that directly affect clinical practice) in seeking to design, configure, and implement blockchain technologies in health care settings. The encountered challenges were typically multidimensional in character, entailing dynamic interacting and often complex technical, social, regulatory, and organizational difficulties that would need to be addressed successfully before the technology is likely to produce significant value for health care.

The final stage of analysis sought to develop a more theoretically grounded account of the observations generated at the second stage analysis, drawing upon different fields of scholarship, including academic discussions focused on blockchain’s *revolutionary* potential; the nature, dimensions, and challenges of innovation more generally; and of health IT implementation in particular (including studies of EHR implementation). The aim was to produce an integrated, theoretically informed, cross-disciplinary understanding of the experience of the health care sector’s engagement with blockchain technologies to date, providing a firmer foundation for appraising its prospects by reference to the underlying nature and form of coordination that blockchain technologies currently enable.

### Theoretical Frame

The theoretical framework for this study draws primarily on 3 different but complementary perspectives that collectively contribute to addressing the project’s overarching research question. First, it draws upon the growing cross-disciplinary literature that has typically emphasized blockchain’s *revolutionary* potential to enable novel forms of social cooperation between strangers without the need for a trusted third-party intermediary. Blockchain’s potential to enable *trustless* cooperation is ultimately attributable to its underlying technological architecture and distributed operation to produce a shared database that is effectively tamper-proof, enabling the replacement of social trust with computational trust in specific contexts. This has prompted some scholars to describe blockchain as a *truth machine* [31]. More recently, however, a more skeptical strand of literature has started to emerge, drawing attention to a wide range of reasons why the technology may fail to live up to its promise [32]. As these inquiries progressed, it became necessary to look to a wider variety of literature to make sense of these real-world implementation challenges. This led to the investigation of a second body of academic scholarship located within the field of *innovation studies*, a different multidisciplinary strand of work that includes a wide range of industry studies that demonstrate that technologies do not arise in the fully developed form [33-35]. Instead, they find that a period of considerable confusion usually follows the emergence of a new technological invention, with little agreement about what its major subsystems should be or how it can best be put together as a product or service, resulting in significant experimentation [36,37].

Health care’s current and ongoing engagements with blockchain can be located within this early experimental phase, situated at the entrance to the so-called *valley of death*. This refers to a *gap* between the development of new scientific knowledge and the establishment of proof of concept but before full development and commercialization [38,39]. Industry estimates suggest that 4 in 5 new inventions are never commercialized because of their failure to overcome numerous *barriers to innovation* encountered during this crucial development phase [40,41]. Innovation studies scholars have identified multiple *valleys of death*, which they have explored from various angles. Some understand the valley primarily as a financing and funding problem that arises after the establishment of a proof of concept. At that moment, significant investment is required to scale up; however, there is considerable technical and commercial uncertainty about its likely success, which thus discourages further investment [34]. Occasionally, this leads to various policy prescriptions, such as government funding initiatives and *translational* centers comprising academic and commercial stakeholders, to help bridge the gap [42]. Others focus on the difficult transition of university research to a product or service brought to market by a commercial firm, particularly in the case of drug development [43].

For the purposes of examining the prospects of blockchain in health care, the most salient strand of this literature is concerned with identifying the *barriers to innovation* that create and contribute to the valley of death. Scholars have classified these barriers as either *internal* to the organization (including factors such as a restrictive mindset, lack of competence, insufficient resources, and an unsupportive organizational structure) or *external* and thus largely beyond the firm’s direct control (including factors such as a paucity of external finance; resistance or lack of support from specific actors, such as customer resistance or an unsupportive government; and various factors which result in a restrictive macroenvironment, such as an undeveloped network and ecosystem, technological volatility that narrows the window of opportunity during which an innovation can be introduced, inappropriate infrastructure, and a restrictive local culture) [44]. However, these largely generic, rather abstract, accounts of various obstacles that must be overcome for technological innovations to largely succeed treat the underlying technology as a *black box* rather than engaging with technological design and specific development challenges in seeking to configure and embed the technology into sector-specific social and organizational systems, processes, and practices [45,46].

Rather than treating blockchain in health care as a closed *black-box*, these theoretical investigations drew upon a third set of analytical lenses situated within academic investigations of health care IT system implementations, particularly studies undertaken from a *social practice* perspective [45,47-49]. These studies highlight the critical role of context, culture, and the values that are brought to bear by medical professionals in clinical environments, identifying clinical consultation as a complex social encounter that occurs within a heavily institutionalized environment [50]. In particular, scholars from these traditions emphasize the role of values in clinicians’ understanding of what constitutes and how they seek to practice *excellent care*. Moreover, they stress the nature of clinical knowledge as *tacit, context bound, and ephemeral rather than codifiable, transferable, and enduring* such that the implementation of health IT into clinical settings often departs substantially from those envisaged by system developers and designers [50]. Accordingly, insights of this kind cast significant doubt on the capacity to hard code norms into technological systems in ways that can be seamlessly integrated into clinical environments, especially when these norms concern the sharing of health data, which is typically and legally understood as highly sensitive and worthy of special protection.

## Results

### Overview

The results of this study are presented in 3 parts, which correspond with the study’s analytical approach outlined in previous sections. First, it briefly describes the health care sector’s engagement with blockchain technologies to date in the United States, United Kingdom, and at the transnational level. Second, it critically reflects upon a series of 6 challenges that must be overcome and 3 normative trade-offs that must be satisfactorily resolved if blockchain technologies are to cross the *valley of death*, transitioning successfully from *invention* to *innovation* in health care settings. Third, it critically discusses key findings by referring to a selection of wider academic literature concerned with organizational cooperation and radical versus incremental technological innovation and reflects upon the promises of patient-controlled blockchain-enabled EHRs before offering some concluding reflections.

### Interrogating Blockchain in Real-world Health Care Contexts

The Motsi-Omoijiade and Kharlamov study [21] revealed that, apart from the Estonian government’s eHealth initiative [22], active engagement with blockchain technology in health care began around 2016, primarily in the United States and, to a lesser extent, in Europe, with several recent initiatives occurring at the transnational level. As of November 30, 2019, their study had identified 128 health care blockchain applications (in which a blockchain *application* is defined as providing a specific functional health care purpose so that a single blockchain system might provide multiple applications) from an English-language website review, over half of which were geographically located in the United States (65 applications), with the United Kingdom as the next most popular site of activity (12 applications) [21]. However, less than half of these had a commercially available blockchain product or service, with most still at the experimental or development stage. Of these applications, a substantial minority (50/128, 39.1%) focused on applications to enable user-controlled access to some kind of personal health data, with a significant proportion concerned primarily with using blockchain to facilitate health care administration (34/128, 26.6%) and support for patient care or health management (primarily in facilitating remote care consultations between patient and clinician located elsewhere) or in the form of *wellness* applications to encourage healthy behaviors (31/128, 24.2%) and for medical research data management (13/128, 10.2%; Multimedia Appendix 2).

A closer examination of the technical and contextual dimensions of these applications and their primary sources of funding revealed that, first, the overwhelming majority of blockchain for health care initiatives had used permissioned or hybrid systems, in which the tasks of sharing and updating the ledger are restricted to those authorized to do so. Second, in both the United States and the United Kingdom, private sector investment and funding have been driving these initiatives, although various government programs support blockchain initiatives, including funding support [51,52]. Although some early initiatives sought to generate funding via an *initial coin offering*, including start-up firms claiming specialist blockchain expertise, these have failed to generate positive returns for investors (and a significant number of initial coin offerings generally were merely vehicles for fraud) [23,53]. More recently, investment funding has been provided by venture capitalists, evidencing their confidence in the technology’s potential value to the health care sector. The commercial and entrepreneurial expertise that they bring to the sector might help the fledgling industry navigate the fraught and uncertain early development phase [54,55]. Third, the current and ongoing focus of interest in blockchain for health care, at least in the US context, has been predominantly at the intraorganizational and business-to-business (B2B) level rather than being primarily concerned with clinical care provision. Much of this activity has primarily concerned blockchain systems for managing *nonclinical* data, as health care providers explore the capacity to harness blockchain technologies to enhance administrative and operational efficiency. Fourth, by comparison, the UK health sector’s experience and engagement with blockchain technology has been far more muted, with only a handful of initiatives, all at the pilot or early-stage implementation phase. These initiatives are primarily for patient care management, with very limited reliance on blockchain functionality. Finally, several promising transnational blockchain initiatives to facilitate medical and health research have been launched in recent years. The following discussion briefly outlines the US and UK experiences (as the 2 most active sites of blockchain engagement in health care) and more recent transnational blockchain initiatives.

### The United States

In the United States, ongoing blockchain activity in health care is coalescing at the intraorganizational and B2B level. These applications are intended to facilitate data sharing between business units within the same umbrella health care organization or within recently established consortia of health care organizations seeking to cooperate in the sharing and management of specific forms of health care data stored via a blockchain-enabled ledger [55]. The primary drivers supporting blockchain development to date appear to have focused on its potential to reduce costs and enhance efficiency in health care administration and operations. These applications are broadly concerned with either health care operations management, primarily in the management of supply chains, or administrative data pooling.

Turning first to health care operations management, considerable blockchain activity has occurred either to streamline and automate internal operational processes or to improve the efficiency and verifiability of supply-chain management [29,56]. Examples of the former include the Coalesce Health Alliance consortium’s development of blockchain to improve the accuracy and efficiency of patient health care claims management across different entities within the Blue Cross Blue Shield ecosystem [57]. Similarly, the US Department of Health’s Office of National Security has built a blockchain providing organizational units with linked, real-time access to a standard set of spend data to improve the department’s procurement process, reportedly generating major efficiency gains [58]. In health care supply chain management, significant blockchain activity has occurred to address the potentially serious risks of a compromised health care supply chain [56]. The Food and Drug Administration has actively encouraged these applications by launching a pilot project (announced in February 2019) to encourage drug supply chain stakeholders to develop digital systems to comply with the Drug Supply Chain Security Act 2013, which mandates the creation of an electronic, interoperable system that can trace and identify distributed prescription drugs across the United States. It attracted 26 participants (including several pharmaceutical supply chain stakeholders [59,60]), generating a variety of projects. These include the MediLedger Project, which sought to build a blockchain network around a tamper-proof ledger of pharmaceutical supply-chain transactions to inform responses to product ID verification requests communicated via a permissioned messaging network [61]. The Pharmaceutical Utility Network has taken a different approach, pursuing a *platform first* strategy [61,62] rather than focusing on a specific *use case*. It is developing an open-source blockchain platform that integrates regulatory requirements (such as the Drug Supply Chain Security Act) to enable those involved in the pharmaceutical supply chain (including pharmaceutical manufacturers, distributors, dispensers, and software vendors) to use the platform’s services to demonstrate compliance with regulatory requirements, seeking to use blockchain as a form of *RegTech*. [63,64] Other supply-chain management blockchains in the health care sector focus on single use cases, often with some RegTech functionality, such as the Clinical Supply Blockchain Working Group’s platform [65], which is now seeking to apply its blockchain-based inventory and event-tracking system to a real clinical trial [66].

Blockchain applications are also being used to facilitate administrative data pooling at the intra- and interorganizational levels. Health care consortia in the United States began emerging in 2018, seeking to use blockchain either to create a single ledger shared between member organizations to serve as a single source of *truth* or to enable network members to create a shared pool of records to which access is controlled and recorded via the blockchain. For example, the Synaptic Health Alliance [67] aims to use a blockchain network to address costs, delays, and inefficiencies in provider directory data (comprising demographic information about physicians and other providers), which insurers are required by federal law to maintain and keep up to date. Each insurer has typically maintained its own directory; however, these often contain inaccurate data, resulting in delayed claims and payment processing. Synaptic’s blockchain pilot seeks to create a common source of *truth* for provider directory data created by pooling and comparing each member’s directory data to create a complete, accurate shared directory.

Similarly, the Professionals Credentials Exchange (ProCredEx) is a consortium of health care organizations seeking to enhance the efficiency of the physician credentialing process [57]. In the United States, when a new physician is hired or begins working for a new payer’s network, the employing health care organization must gather a wide variety of certificates and credentials, a process that takes up to 6 months (sometimes longer), with physicians often required to supply evidence of their professional credentials to a dozen different organizations or more, which must be repeated for all physicians every 2 years by each organization [29]. The ProCredEx blockchain network enables organizations to contribute their credentialing records (essentially, a digital asset) to a shared pool, effectively creating a members-only digital asset exchange so that members can access verified credentials and actively contribute credentials information that other members can acquire. The shared ledger tracks asset ownership, exchange, and use and forms a basis for asset reputation. Buyers of these information assets finance the platform, whereas curators of the assets receive payment for their contributions, as do partners who help facilitate the use of the blockchain network [68,69].

### The United Kingdom

In comparison with its US counterpart, interest in blockchain in UK health care has been considerably more limited [70]. The UK blockchain use cases have been directed at patient care management largely outside the clinic, in which secure and auditable data sharing remains the central functionality provided by blockchain technologies. Unlike the United States, UK health care is delivered primarily via a publicly funded National Health Service (NHS), in which NHS hospital treatment and primary care are free at the point of use to UK residents. Decisions about the commissioning of services, including the use of new technologies, are taken primarily at the local level by entities known as clinical commissioning groups or exceptionally at the national level by NHS England, which is informed by guidance issued by the UK’s National Institute for Health and Care Excellence [71]. The NHS Long Term Plan states that the NHS will seek to “make better use of data and digital technology” [72] as part of the NHS digital transformation agenda; however, it makes no explicit reference to blockchain technologies. However, the NHS England’s Director of Digital Development has been reported as having a general interest in considering blockchain as one of a variety of technologies that the NHS will explore [73].

Nevertheless, NHS England has commissioned Dovetail Labs to develop a novel digital application to enable different members of a multidisciplinary team working with type 2 diabetes patients to share health information. The application operates via a distributed ledger that logs patient consent to share digital data along with user authentication, data access, and transfer records [74,75]. In addition, at least two local clinical commissioning groups have commissioned small-scale digital applications that are described as using blockchain technology. Hence, a partnership between Guardtime and Instant Access Medical and Healthcare Gateway has produced a comprehensive blockchain-supported personal care record platform (called *MyPCR*). The platform is intended to assist patients in managing chronic long-term health conditions by issuing digital alerts to help them adhere to their personalized treatment plan or personal care pathway via a smartphone app, granting patients complete access to their personal care pathway while generating an automated tamper-proof record of health data provenance and integrity that is compliant with data protection legislation [76]. Similarly, MedicalChain has launched a blockchain pilot enabling patients to create a free wallet to manage access to their digital health records, enable general practitioner video consultations, and enable them to pay for services using cryptocurrency (with users incentivized to pay for telemedicine services with Medicalchain’s MedTokens) [77,78].

### Transnational Blockchain Systems

At the transnational level, a rather different kind of blockchain application has emerged, which enables network members to run secure but privacy-preserving computational analyses on the data of other members to improve their algorithmic models for research purposes while the data never leaves the host organization [79]. For example, members of the Machine Learning Ledger Orchestration for Drug Discovery blockchain network, comprising 10 large pharmaceutical companies, 5 technical partners, and 2 universities, use blockchain and federated learning to train an algorithm to identify a rare mutation in cancer, enabling pharmaceutical companies to collaborate on therapeutic drug discovery. Each pharmaceutical company can, via the network, run the machine learning algorithms of their academic partners on each other’s data sets. The data remain at their local site so that proprietary information from one data set cannot be leaked to another. Nonsensitive algorithmic models are exchanged between the members, which are then collectively consolidated to improve the predictive performance of the algorithmic models by leveraging all the data across the federation. A permissioned blockchain tracks and logs all operations taking place on the federated data using a software framework (Substra) for enabling the execution of distributed machine learning tasks in a secure way. The value of collaboration is particularly evident in attempts to develop drug treatments for rare diseases: each individual organization will have too few patients with a given rare disease to undertake statistically significant analysis; however, by pooling access to their data to enable federated learning, collaboration without data sharing becomes possible. Similarly, the AI Centre for Value-Based Healthcare consortium has created a federated learning network with patient data from 4 hospitals and 3 universities, allowing research partners to train algorithms on the federated data set. Collaborations of this kind, which use blockchain networks to facilitate privacy-preserving, federated data access, offer considerable promise to advance medical research [29]. Health care blockchain industry expert Robert Miller [80] observes the following:

Health data is inherently sensitive, and thus demanding of privacy. Yet at the same time health data is inherently statistical, and thus holds within it insights that could improve health for all. The promise of federated learning is to unlock these insights without compromising on privacy. Moreover, federated learning will enable us to assemble more data than ever in federated networks, leading to better algorithms and ultimately better outcomes.

### Challenges for Blockchain Take-up and Implementation in Health Care Settings

#### Overview

We have seen how the health care sector is beginning to develop viable blockchain applications for specific health care purposes and contexts to establish proof of concept, which are yet to be widely implemented at scale [58]. This invariably invites questions about the prospects of blockchain’s sector-wide take-up, implementation, and diffusion, particularly given the limited success of health IT adoption more generally [81]. To begin addressing these questions, a set of 6 interrelated *implementation challenges* associated with seeking to implement and integrate blockchain into real-world health care contexts were identified using the methodological approach described above. Of these, 3 challenges were already being encountered in seeking to use blockchain technologies to facilitate the sharing and collaboration of health care data for administrative and research purposes, namely (1) organizational commitment, (2) interoperability and standardization, and (3) internal governance. A total of 3 additional emerging challenges are likely to be particularly acute if, in the future, sustained attempts are made to introduce blockchain technologies into clinical settings for which only modest experimentation has occurred to date, namely (4) data security and integrity, (5) quality and safety, and (6) truth and immutability (Textbox 1).

In addition to these implementation challenges, 3 further *normative tensions* or value conflicts invariably arise in configuring blockchain technologies to meet specific functional requirements in health care contexts, which were also identified (Textbox 2). Accordingly, acceptable compromises between competing objectives and values must be identified in an attempt to apply blockchain to real-world settings that can be designed into the software and system architecture and implemented into specific organizational contexts [82].

**Blockchain in health care implementation challenges.**

**Implementation challenges**

- Organizational commitment
- Interoperability
- Internal governance and standardization
- Data security and integrity
- Quality and safety
- Truth and immutability

**Normative tensions requiring resolution in applying blockchain to health care.**

**Normative tensions**

- High performance and scalability while providing adequate security
- Providing transparency and accountability while ensuring due respect for privacy and confidentiality
- Establishing computational trust while securing adequate social trust between network participants

Common to each of these challenges and normative tensions is their multifaceted, dynamic, context-sensitive, and complex character, comprising both technical and nontechnical dimensions. Moreover, addressing these challenges typically requires internal organizational adaptations *and* changes external to the organization because of the inherent character of blockchain as a *process* innovation [83], particularly when used to facilitate trustworthy data sharing beyond organizational boundaries. Each of these challenges is outlined below, beginning with 3 near-term challenges, followed by a brief discussion of 3 further challenges that are likely to be particularly acute if blockchain applications are applied in clinical contexts.

#### Organizational Commitment

The decision by any organization to adopt a new IT system is not made lightly. Making a commitment to experiment with a technology to establish proof of concept as a bounded, time-limited project is likely to be considerably easier for an organization than committing to implementing a novel technology on an ongoing basis, particularly for health care organizations [84]. Difficulties associated with eliciting the funding and commitment necessary to move a technological invention beyond the proof-of-concept stage are one of the characteristic features of the so-called valley of death described above. For organizations, this requires a compelling business case demonstrating that such a move will generate significant, sustainable net gains (such as increased revenues, reduced costs, or better-quality patient care) to justify the substantial investment and risks associated with doing so [85,86]. Given that the nature and magnitude of benefits of blockchain for health care remain largely unproven, and experience has shown that implementing health IT programs involves a great deal of time, money, and effort, making such a case will be difficult [9,81,85,87]. When proposed to support administrative functions, such as supply-chain management, the cost of blockchain adoption and implementation may be difficult for an organization to justify, as the expected benefits take the form of enhanced compliance and, hopefully, lower costs and reduced risk, all of which are difficult to quantify. For example, consider difficulties in quantifying the value gained from using blockchain-enabled pharmaceutical supply chains to reduce the risks associated with the production of fake medicines whose scope and prevalence are not well-known, and alternative cheaper technologies which may be more attractive [29]. Organizations willing to attempt blockchain implementations will also need to join a suitable network of participants willing to align and collaborate over a common interest in a particular kind of health care data via a blockchain network and avoid generating significant user resistance from staff. Neither task is likely to be easy [30,32,88-90].

Nonetheless, the proliferation of health care consortia in the United States, throughout 2019 and early 2020, seeking to develop blockchain-based collaborations over administrative data suggests that a variety of health care organizations increasingly recognize the potential value of blockchain-based data sharing for administrative and research purposes. The use of blockchain-enabled data sharing to support highly labor-and time-intensive administrative processes (such as provider credentialing) could significantly enhance administrative efficiency and reduce workloads. As one leading commentator has observed, almost every insurance company and pharmaceutical company has announced participation in a cooperative data-sharing health care consortium, including health systems, IT companies, and nontraditional health care companies such as PNC Bank and Walmart [91]. However, blockchain applications that have gained the most organizational buy-in have either avoided sharing of patient data, focusing instead on collaboration over administrative data, or have involved research collaborations that share and manage patient data in a privacy-preserving manner.

This points to a further hurdle that is likely to arise in eliciting organizational commitment: the need to ensure that blockchain applications comply with applicable legal and regulatory requirements, which are especially demanding in relation to clinical applications. Legal requirements that apply to patient-related health care data and the broader regulatory environment in which health care is provided are substantial, complex, and often difficult to navigate, both in the United States and Europe. Accordingly, ensuring that blockchain-enabled *clinical* applications demonstrably comply with applicable laws will be very challenging [9,92]. That said, blockchain advocates place considerable optimism in *smart contracts*—computer programs that automate the verification, execution, and enforcement of certain terms and conditions of an arrangement, built on top of a blockchain system that enables a distributed ledger to function as a distributed computer [8,93-95]. Accordingly, blockchain systems in combination with smart contracts could operate as a form of *RegTech* through which compliance with legal and other regulatory requirements can be *hardwired* to execute automatically. However, whether these technologies will live up to this potential, given the messiness and unpredictability of real-world contexts, remains unknown [96,97]. Overcoming each of these barriers to organizational commitment presents formidable challenges and taken together, generate major obstacles that lie in the path of successful blockchain innovation and diffusion in health care [98].

#### Interoperability and Standardization

Another significant obstacle that continues to frustrate health care data-sharing efforts is the lack of interoperability within and between health IT systems [99]. This is equally true for blockchain-based data management systems. HIMSS explains that, for the purposes of a health data ecosystem, interoperability is “the ability of health information systems to work together within and across organizational boundaries in order to advance the effective delivery of healthcare for individuals and communities.” HIMSS [100] notes that:

In the healthcare space, interoperability is important across systems and organizations for information to flow seamlessly between actors like patients, doctors, hospitals, payers, etc. However, it has been one of the hardest challenges healthcare has faced as vendors, providers, policies, payers and patients have, at times, set roadblocks up and created misaligned incentives to achieve true exchange of data between disparate systems. Realizing the benefits of blockchain technology depends on the associated network of healthcare organizations being able to share data and collaborate via the blockchain. These net-new requirements increase the scope of interoperability challenges that pre-dated blockchain. When systems are not interoperable, the cost of verification and networking of the information is very high. The same is true for the cost of settlement and reconciliation of the transactions of information.

To maximize the capacity for data sharing, *full* interoperability between blockchain systems is needed; however, achieving this remains a very long way off and arguably will forever remain an impossible dream [101]. Interoperability in relation to health care IT can be understood at 3 levels. First, foundational interoperability allows data exchange from one IT system to be received by another without needing the receiving IT system to have the capacity to interpret that data. Second, structural interoperability allows the movement of health care data from one IT system to another such that the clinical or operational purpose and meaning of the data are preserved and unaltered, which is enabled by defining the structure or format of data exchange. Third, semantic interoperability refers to the ability of 2 or more IT systems or elements to exchange information and use the information that has been exchanged [102], requiring both the structuring of the data exchange and the codification of the data (including vocabulary) so that the receiving IT systems can interpret the data. Critical to the achievement of interoperability is the shared use of common standards (that are either mandated or very widely accepted and adopted) at the relevant level. To this end, important and ongoing efforts are being made to establish international standards for health care data and IT architecture to enable all 3 levels of interoperability for health care data sharing, including the work of standard-setting organizations such as Integrating Healthcare Enterprise International [103] and Health Level Seven International [104,105].

The need for interoperability may help explain why the blockchain use cases with the greatest prospects of success in the near term are largely concerned with sharing *back office* functions and associated records, such as credentialing and provider identity verification, and claims management and billing rather than the sharing of patient data for clinical purposes [106,107]. Although these applications entail interorganizational record sharing, which may well be in different formats and structures, the substantive content of the records themselves is likely to have a high degree of *semantic* interoperability that tends to avoid subjective evaluations and is not readily prone to misinterpretation or misunderstanding. For example, the ProCredEx consortia enable the pooling of records concerning whether a particular individual possesses particular professional qualifications; these records can be readily shared between participants without any serious likelihood that the meaning of those records will be misinterpreted by other members [57].

In other words, blockchain-based record sharing among multiple participants is more likely to succeed if the underlying data are highly stable and readily verifiable as evidencing the *truth* of the underlying phenomena that the data purports to represent (eg, such as whether a clinician has obtained a university degree from an approved medical school), and the consequences of an error or data inaccuracy are not safety-critical. However, experts worry that, as multiple blockchains for health care networks emerge supporting a wide variety of applications, they might not be interoperable *with each other*. This could seriously undermine the value and benefits of blockchain-enabled solutions [108,109]. For example, an organization participating in a pharmaceutical supply-chain blockchain may find that it is not interoperable with a clinical trial blockchain network that it also wishes to join, significantly reducing the potential value it might otherwise derive from using blockchain. These anticipated challenges reflect the observations by Greenhalgh et al [98] that a common but significant health care infrastructure challenge in many countries lies in enabling state-of-the-art individual technologies to interface with (but have often been designed with little awareness of) a legacy infrastructure and restrictive regulatory standards, all in the context of a complex, fast-changing, unpredictable, and underfunded service environment.

#### Internal Governance

A feature of the growing number of health care consortia seeking to use blockchain technologies to selectively share data and collaborate at the B2B level is that member organizations are often conventionally regarded as competitors [110]. If these novel sociotechnical collaborations are to succeed, a clear set of agreed norms and arrangements to govern the terms of their collaboration will be essential, without which stable cooperation across the network is unlikely to be viable in practice. In open, permissionless blockchains, including Bitcoin and Ethereum, disagreements over proposed changes to the technical architecture have led to highly publicized disagreements, reflecting the different political viewpoints and motivations of participants, which often lead to the fragmentation of the blockchain network in the form of a *fork* in the ledger [111-114]. Although the overwhelming majority of health care distributed ledger technology applications use hybrid enterprise or permissioned systems, they face internal governance challenges that may be no less fraught than those that have arisen in open, permissionless blockchains [115]. However, establishing a durable internal governance framework that commands widespread acceptance by members and participants across the network (including potential new members and participants) will be especially challenging, which is discussed more fully in the following sections in relation to the challenges of *coopetition* [116].

#### Data Security and Integrity

Although the most promising engagements with blockchain in health care contexts have hitherto largely avoided the clinic, many industry experts believe that as the technology matures, clinical applications entailing collaboration over patient data are likely to emerge [117]. A further 3 challenges are already being encountered within the health care sector in seeking to engage with blockchain technologies, but which are likely to be particularly acute if blockchain applications are applied in clinical contexts. Chief among them are the challenges associated with data security and integrity. For any blockchain-based system, the desired level of data security must be identified, established, and maintained across the system’s architecture and operation. For patient data, questions about data security are primarily informed by their size and high sensitivity. Accordingly, it is widely accepted that patient data are best stored *off chain*, for example, in a relational database with the shared ledger merely storing metadata together with pointers to where the actual patient data resides and hash codes to verify the integrity of the off-chain data [82,118]. By incorporating technological mechanisms for identity management and access control into blockchain systems, the on-chain record can also record when, whether, and by whom the relevant linked data are accessed. This approach is reflected in what HIMSS refers to as the principle of *minimal sufficiency* to on-chain data, which it advocates as best practice, stating the following [92]:

Blockchain technology was not designed to be a storage mechanism for data and should not be leveraged as such, for security, privacy, compliance and performance reasons. Information added to the chain may be transparent to permitted network participants and difficult to remove without affecting the entire chain. Therefore, we strongly recommend that regulators and policymakers promote that organizations leveraging blockchain-enabled solutions employ a “minimal but sufficient” strategy for the data that should be included on-chain. This strategy should be guided by the use case’s data needs, and implementers should keep in mind not only the privacy and security risks but also the performance of blockchain transactions when deciding the amount of data and/or personally identifiable information (PII) included on the chain. Whenever possible, privacy-enhancing technologies should be used to secure private data on the blockchain.

However, by storing patient data offline, the blockchain ledger cannot ensure data security. Although there are various measures, including encryption, that can enhance off-chain data security, these are separate and distinct from blockchain’s technological protections for securing on-chain data. Given that one of blockchain’s most significant *promises* lies in the iron-clad security it purportedly brings to on-chain data, the inability to extend that protection to the data stored off chain seriously limits the technology’s capacity to deliver on this promise. In addition, a frequently overlooked and neglected issue in the blockchain literature is the problem of *data leakage* or *escape*. Although blockchain offers the technological capacity for fine-grained, auditable data sharing through technological access controls that protect the privacy and confidentiality of stored records, blockchains do not address the possibility that, once data are revealed, those with access will generally be able to copy and extract the data and store it perpetually. As Finck [93] warns, the purposes of blockchain projects can be completely undermined when data escape is possible, particularly in circumstances where data are sold once, and the buyer can then resell or manipulate the data set at will.

#### Truth and Immutability

A critical feature emphasized by blockchain advocates is the technology’s capacity to create a single, immutable, shared authoritative record, reflected in descriptions of blockchain as a *truth machine*. However, the validity of this claim assumes that the data recorded on the blockchain has *integrity*—meaning that they are accurate, up to date, and comprehensive. Although often discussed in terms of providing assurance that the data have not been tampered with or subject to unauthorized alteration [119,120], data integrity also requires that the data faithfully and accurately represent the underlying real-world phenomenon they purport to represent. For example, if a health care record is labeled as representing a particular item, such as an X-ray taken of patient Y on Z date at location P and stored at an off-chain location Q, then it must represent precisely that. This also requires (among other things) that blockchain consistently keeps transaction information associated with the correct person. Although considerable work is being devoted toward identifying universal identity management solutions for blockchain-based systems, this remains an unresolved challenge. Questions concerning how blockchain can ensure that the records appended to the blockchain ledger are accurately tethered to and reflect the underlying reality that they purport to be associated with remain surprisingly overlooked (or perhaps conveniently ignored) in the existing blockchain literature. In health care, these challenges are particularly acute if data are to have clinical relevance. However, they sit uncomfortably with developers’ and entrepreneurs’ claims that blockchain will enable the creation of accurate and comprehensive patient records that draw data directly from a patient’s wearable device, which is gathered via sensor technologies, without mechanisms that guarantee the accuracy, veracity, and reliability of data thereby collected [21]. Although sensor technologies can help establish direct connections between the digital and physical world in a secure manner, sensors can be easily tricked (even if tamper-proof hardware is used); thus, conventional social trust is needed to trust the veracity of the underlying data. This calls into question the value that blockchain purports to offer in the first place. In the context of supply chain management, Wüst and Gervais [119] refer to the following:

The inherent problem of the interface between the digital and the physical world. A human, or some machine under the control of a single writer, typically is required to register that a good has arrived in a warehouse and, if for example, its quality is appropriate. If there is no trust in the operation of these employees, then the whole supply chain is technically compromised as any data can be supplied by a malicious writer. If, on the other hand, all writers are trusted, a blockchain is not needed as a regular database with shared write access can be used instead.

Although the accuracy of health care administrative data may not always be safety-critical, there is little value in a blockchain that maintains a tamper-proof record of inaccurate or poor-quality data. Accordingly, descriptions of blockchain as a *truth machine* appear somewhat overstated [31]. Data integrity is a necessary prerequisite for establishing trustworthiness and providing a reliable basis for decision-making. Houlder [121] explains the following:

Data security is often equated with protecting data confidentiality: but it is data integrity that must be protected – and this requires ensuring that the data is accurate, up-to-date and complete. Blockchain only protects what arrives at blockchain. From an immutability and transparency standpoint, if that data is compromised or of poor quality before it reaches the blockchain you end up with garbage in, garbage out, where you’re protecting garbage on the blockchain. Unless the data has the integrity and quality needed, blockchain will not realise its potential.

The yet-to-be-resolved challenge of assuring data integrity may help explain why the handful of patient-facing blockchain applications offered through the NHS (in partnership with private technology providers) have largely confined blockchain functionality to storing records of patient consent and providing computational assurance concerning when a patient has accessed (and securely copied) his or her NHS records.

In addition, the application of blockchain to health care will entail human interaction and engagement. Even if users are provided with high-quality training, help, and guidance, mistakes will be inevitable. However, identifying and correcting errors generates new challenges, particularly given that blockchain ledgers cannot be retroactively altered. Although blockchain’s tamper-proof character prevents certain kinds of mistakes and problems that arise in relation to conventional centralized databases (such as inadvertently overwriting data stored on the database), they will inevitably introduce new ones. This is particularly so given the complex and dynamic nature of health care settings and the multiple intersecting and sometimes conflicting interests, rights and obligations, expectations, and anxieties they typically implicate. In other words, the vagaries of human behavior and decision-making in real-world health care contexts could prevent blockchain’s expected benefits from being fully realized.

#### Quality and Safety

Data integrity is but one element of the larger, multidimensional challenge of ensuring that blockchain systems can be implemented in health care without compromising quality and safety, particularly when they interface with and operate within clinical contexts. Although the safety implications arising from the digital transformation of health care have been well-documented [122], patient safety has received relatively little attention to date, perhaps because of the relative scarcity of clinical blockchain use cases being trialed and implemented. Patient safety issues include the need to ensure that blockchain systems that affect clinical workflows and environments are subject to rigorous testing, validation, and independent evaluation before their introduction into the clinic. However, in the US health care context, no systematic attention appears to have been given to the technical robustness, safety, or ethical dimensions of blockchain implementation. Rather, health care blockchain projects appear to move from proof of concept through to implementation without necessarily being subjected to formal testing and validation to provide assurance of the system’s robustness, resilience, or clinical safety. Accordingly, health care blockchain expert Heather Flannery argues that these projects should be understood as experiments to learn and discover the kinds of conditions that produce the desired results; although these conditions are partially technical, in her view, most are clinical, social, economic, legal, ethical, and governance-related. Hence, Flannery argues that these projects should be undertaken as studies subject to proper research protocols as the only ethically viable way to move past the point of experimenting with the technology [123,124]. In contrast, regulatory oversight of health IT systems in the United Kingdom is more developed. Thus, the Health and Social Care Act 2012 requires the development and implementation of clinical risk management processes to ensure patient safety with respect to the manufacture of new health IT systems, or the modification or decommissioning of an existing system, and their deployment and use [125,126].

### Normative Conflict Requiring Satisfactory Resolution

Although the above 6 challenges might be overcome in time, several further challenges rooted in inherent tensions between desirable qualities or functional requirements that arise when configuring blockchain applications may prove more intractable. Although technical blockchain experts have identified various *design trade-offs* when building blockchain systems for specific purposes [82], reflecting the *micropolitical nature of software choices* [98], the following 3 are particularly apposite to health care settings:

#### Performance and Scalability Versus Security

Ideally, an IT system would provide high levels of security and resilience for the system and the data it stores and generates while offering high-speed performance at scale. However, achieving these objectives in blockchain systems is technically impossible (on currently available technology) because of inherent trade-offs in functionality. There is a loose *trilemma* in the design and configuration of blockchain systems such that they can have at most two of the following three properties: (1) decentralization, (2) scalability, and (3) security. The more computationally demanding and time intensive the consensus protocols for validating transactions [127] before being appended to the ledger and the greater the degree of decentralization across the network, the greater the ledger’s security and resilience against attack but the slower the performance in terms of speed and throughput [128]. In addition, as blockchains ledgers are distributed, they entail high storage costs, precluding large data from being effectively stored on the blockchain. As already noted, although blockchain can be used for access control (and auditing), large data must be stored *off chain* [129]. This generates the need to assure off-chain data security; simply using a blockchain to manage access does not thereby offer the security of the stored data, pointing to an inherent tension between the desired values of scalability versus security in their design and operation [130].

#### Transparency Versus Privacy

The transparency of all transactions appended to the ledger is a critical feature of open, permissionless blockchains, contributing to the ledger’s trustworthiness. However, this level of transparency is fundamentally at odds with the private and confidential nature of patient data and other health-related personal information. Accordingly, there is an inherent tension between the need to respect privacy and confidentiality and the design and operation of open blockchain networks that are transparent to the world at large [93]. Under the European Union’s General Data Protection Regulation, data collectors and processors must not collect or process personal data without a lawful basis, which includes, but is not limited to, consent by the data subject. In common law systems, additional legal obligations attach to information acquired in circumstances of confidence by clinicians and other care providers, including information communicated by patients to doctors during clinical encounters. At the same time, health care organizations typically seek to keep details of their business records confidential. As already indicated, the *minimum sufficiency* principle of information collection and storage is considered best practice in health care IT design and operation so that health care blockchain systems have hitherto largely taken the form of *permissioned* systems, thereby limiting *read* access to the ledger to those authorized to do so, with health care data being stored off chain and only the hashed metadata stored on chain.

#### Computational Trust Versus Social Trust

Although the hyperbole associated with blockchain technologies has begun to subside, early industry activity and engagement with blockchain appears largely to have been *technology lead*, arguably motivated by the desire to engage with the latest cutting-edge technology [131] without either a deep understanding of the technology or a desire to meet a specific need that blockchain might usefully address. As the technology has begun to mature, so too have academic investigations, with more recent academic critiques arguing that conventional database structures and systems will often provide cheaper, faster, more sustainable, and scalable approaches to specific real-world data access, management, and storage needs [119,132]. Several scholars have sought to identify the circumstances in which blockchain technologies have a genuine prospect of addressing real-world problems for which conventional centralized, networked databases are inadequate. For example, Wüst and Gervais [119] argue that if either a trusted third party who can verify transactions is always available on the web or if all writers mutually trust others, then a conventional database with shared write access is likely to be preferable. However, if the writers of the ledger do not trust each other, a permissioned blockchain may provide a viable solution. Their insight resonates with, and may help to explain, the recent emergence of consortia of health care organizations who compete with each other in many respects but recognize that they share a limited common interest in relation to specific kinds of data and forms of data sharing. In these circumstances, member organizations may not fully trust each other, and a permissioned blockchain may provide a technological solution to address this trust deficit.

Permissioned systems (including so-called *enterprise* systems) have hitherto been favored in health care blockchain applications. They appear to offer the greatest opportunities for generating value from network effects arising from the creation of a shared, trustworthy transactions ledger between a limited number of authorized participants, enabling members to benefit from effectively sharing elements of a *back office* administrative system (ie, shared records management in a single shared ledger). However, the security and integrity of the shared record will only be as strong as its weakest link, giving rise to what may be called the *computational trust* paradox. On the one hand, the use of computational consensus mechanisms to verify transactions and maintain a single shared ledger across a distributed network of computers obviates the need for each participant to maintain their own ledger and reconcile their ledger with that of other participants, generating significant administrative efficiencies. On the other hand, this means that each participant must trust the integrity, quality, and security of the participant’s records, including the accuracy of the information contained in those records. This relies, in turn, upon the integrity of the practices and processes underpinning the events and transactions that each record purports to represent (subject to those records being validated by the network and appended to the shared ledger). By pooling their records, each participant is thereby exposed to the vulnerabilities of the record keeping and information security practices of their fellow participants. A paradox arises because, although blockchain systems offer the possibility of coordination with others *without* the need for social trust in those others by relying instead on *computational trust*, when these systems take the form of permissioned ledgers, this is likely to *enhance* the need for conventional (social) trust in the practices and systems of other network members. Hence, reliance on computational trust serves, ultimately, to heighten the importance and need for social trust to reap the gains from cooperation [133]. A leading industry expert [121] commented as follows:

If you have a consortium of 10 organisations and one of them is a kind of weak link...in terms of they’re connecting to the blockchain and pulling information off the blockchain, or in interactions that are enabled by blockchain, and putting information in their own enterprise systems which are insecure and a breach occurs, it impacts everyone...you’re not just worried about securing your own organisation, you’ve got to make sure that everyone connecting to that blockchain is adequately secure.

## Discussion

### Overview

The above findings draw attention to the number and variety of formidable, multidimensional challenges that must be satisfactorily addressed if blockchain technologies are to traverse the valley of death. Nevertheless, this study also shows how early engagements with blockchain in health care have begun to demonstrate that these technologies *can* enable and facilitate novel, valuable forms of health care data sharing and cooperation in real-world health care settings under specific circumstances. The following discussion critically reflects upon these key findings by reference to 3 quite different strands of academic literature: first, it examines the character and tensions inherent in these incipient forms of blockchain-enabled cooperation; second, it draws on insights from innovation studies to consider the character of blockchain as *radical* or *incremental* health care technologies; and third, it evaluates the prospects of blockchain for health care’s *favorite use case*, that is, to enable *patient sovereignty* over EHRs while overcoming the currently siloed approach to patient data.

### The Blockchain in Health Care Promise: Rhetoric, Reality, and Blockchain-Enabled Coopetition

Recent health care blockchain initiatives have successfully demonstrated how technology can facilitate limited, purpose-specific forms of cooperation through the shared pooling of data to solve common problems. These incipient forms of cooperation involving data sharing, which consortia of health care organizations are actively seeking to develop, rely upon interactions that organizational studies scholars refer to as *coopetition*. Although the interaction between firms conventionally focuses on either of 2 contrasting and antagonistic logics—competition (traditionally defined as the conflicting and rivalrous relationship between firms) on the one hand and cooperation on the other [134]—the actual interaction between firms can involve cooperation in some activities while competing in others [135]. As these coopetitive relationships entail 2 diametrically opposing logics of interaction, they are inherently complex, generating tensions between and within organizations [136,137]. In particular, each organization faces conflicting incentives: a desire to cooperate through the pooling of resources to generate shared benefits on the one hand and the incentive to capture private benefits for themselves on the other [137,138]. Several theories have been offered, claiming to predict the effect of coopetition on firm performance [137,139], including transaction cost theories. Transaction cost theories highlight the difficulties of fostering trust between parties in a coopetitive relationship, as each participant has incentives to opportunistically behave while facing high levels of uncertainty [140]. This creates barriers to open collaboration, making it difficult to develop the necessary level of trust needed for common projects to succeed. On the other hand, several scholars have noted that the inherent tension between cooperation and competition arising within coopetitive relationships can, if managed efficiently, create shared value and generate beneficial outcomes for each party [137]. On this view, the key to successful coopetition is effective management of the coopetitive tensions within the relationship that nurtures and maintains an appropriate balance between competition and collaboration. Various insights from this literature help illuminate the challenges likely to arise within health care consortia comprising organizations that would otherwise regard themselves as competitors seeking to share health care data via a permissioned blockchain network. The configuration of access rules within a permissioned blockchain system offers a potentially novel mechanism through which *social distrust* might be replaced by *computational trust*, thereby enabling new forms of cooperation at the B2B level that might not otherwise occur, as the trust gap is too difficult to overcome.

That said, the need for strategies to successfully manage the tension between organizations within the network highlights the importance of a blockchain network’s internal governance arrangements, particularly in establishing and maintaining rules concerning changes to the network’s architecture, protocols, rules of admission, and incentive mechanisms. Details about the internal governance arrangements that underpin the various blockchain consortia that have emerged in recent years are not publicly available because of the confidential nature of the underlying partnership agreements. Nevertheless, one would expect that formulating and maintaining a set of governance arrangements that will prove durable and resilient over time is likely to be extremely difficult, as the interests of participants are not *wholly* aligned and hence, difficult to settle in advance. These internal governance arrangements must identify how critical decisions about, for example, the structure of economic incentives or rewards for participants to engage in the consensus protocol, how the network will be maintained, and how other changes to the network’s structure and operation will be made and by whom. Potentially conflicting interests may not be readily apparent at the time of the network’s inception but may subsequently arise as organizational needs, the larger industry context, and other external factors inevitably change over time. However, perhaps it is only when the *rubber hits the road* in the context of specific disagreement between network participants that the critical need for robust governance structures that command the respect and allegiance of the network’s members becomes apparent.

Finding an appropriate but flexible equilibrium between the need for social trust between network participants on the one hand and *hardwiring* computational trust into the technical architecture and operation of the blockchain system will be a very tough nut to crack. It remains an ongoing and continuing challenge for the foreseeable future, at least until blockchain in health care achieves a level of maturity in which a widely shared set of agreed norms concerning the core requirements of good blockchain governance can emerge. Furthermore, these networks are likely to encounter what has been referred to above as the *computational trust* paradox; by seeking to rely on computational trust to provide a basis for enabling cooperation between firms, this may reinforce and accentuate the need to nurture and maintain social trust between the parties in domains in which computational trust cannot be secured.

### Permissioned Blockchains in Health Care: Incremental or Radical Innovation?

Early engagement with blockchain technologies in health care contexts has largely used *permissioned* blockchain systems (including so-called *enterprise* blockchains) rather than open, permissionless systems that many blockchain advocates believe will revolutionize social cooperation between strangers without the need for conventional trusted third-party intermediaries, such as banks and governments. Although the blockchain for health care promise has not typically emphasized the role of permissionless blockchains, a significant *revolutionary* narrative is evident in claims that blockchain technology will *disrupt* health care [17]. Within this narrative, blockchain is portrayed as enabling radical patient empowerment achieved via hard-coded access controls, securely facilitating the seamless exchange of patient records currently stored in disparate data silos while enabling patients to exert fine-grained control over who is granted access to those records, wherever located, and on what terms [141,142]. It is blockchain’s potential to overcome the many existing technical, legal, and bureaucratic obstacles that obstruct the free flow of health data while preventing patients from exerting control over access to their own health care records that is typically highlighted rather than emphasizing the role of permissionless blockchains in achieving this vision.

The health care sector’s focus on permissioned (rather than permissionless) blockchain systems to date will disappoint radical blockchain enthusiasts such as Bruce Schneier, a well-known computer security expert. Schneier claims that permissioned blockchains are “completely uninteresting” as they are no different from centralized databases in relying on conventional forms of trust to facilitate social cooperation [143]. However, his perspective fails to recognize that even open, permissionless blockchains ultimately and invariably rely on social trust to support their internal governance arrangements [112,113]. By enabling participants to rely on computational trust mechanisms to enforce the explicitly agreed terms of their cooperation, permissioned blockchains could facilitate novel forms of cooperation, leading to valuable and potentially transformational change.

The transformational potential of permissioned blockchain systems can be illuminated by reference to a distinction drawn by technology management researchers between *inventions*, which refers to an “idea, sketch, or model for a new or improved device, product, process or system” and *innovations,* which, in the economic sense, arise “only with the first commercial transaction involving the new product, system or device” [144]. Within this literature, a further distinction is commonly made between *incremental* and *radical* innovation. Incremental innovations introduce relatively minor changes to an existing product, exploiting the potential of the established design (although they may entail considerable skill and ingenuity) [145-148]. In contrast, radical innovations are based on different engineering and scientific principles, potentially opening up whole new markets and applications [146,147,149]. This distinction is considered to have important competitive consequences: incremental innovation tends to *reinforce* the capabilities of established organizations, whereas radical innovation forces them to *ask a new set of questions*, draw on new technical and commercial skills, and use new problem-solving approaches [36,146,148,150]. Hence, radical innovation often creates great difficulties for established firms [148,151-153]. As the late Clayton Christensen famously argued, radical innovation can *disrupt* the entire industry as incumbent firms lose out to smaller firms with fewer resources who use these radical innovations to deliver superior functional performance, eventually leading to their adoption by the incumbent’s established customers [154].

It is too early to assess whether permissionless blockchains will ultimately form the basic technological architecture underpinning a dominant design for particular kinds of health care applications. It is not possible to reliably predict whether they will come to be regarded as incremental or radical innovations or, indeed, whether they come to be understood as innovations at all or merely just an interesting invention with little real-world utility. However, the preceding insights suggest that we cannot assume that the use of permissioned rather than permissionless blockchains in health care necessarily implies that they cannot subsequently establish themselves as *radical innovations*. Bruce Schneier’s dismissal of permissioned blockchains overlooks the possibility that they may offer particular functional properties capable of meeting a very specific sectoral need. In functional terms, permissioned blockchains are something of a hybrid—analogous to centralized databases insofar as participation and access require authorization from a network controller but can mathematically provide the verified security and automated audit functionality of permissionless blockchains without the heavy computational expense. Accordingly, they might prove *radical* in their effects [155], creating shared value by facilitating new forms of coopetitive collaboration between health care organizations while generating significant efficiencies.

### Blockchain-Enabled EHRs and the Promise of Patient Data Sovereignty

Mention has already been made of frequent references in both academic and industry literature to the potential of blockchain-enabled EHRs to *revolutionize* health care. According to this vision, blockchain operates as a form of *middleware*, facilitating the flow of data between independent IT systems, thereby integrating disparately located silos of patient health care records while enabling patients to exercise fine-grained but automated control over their EHRs via *smart contracts* that automate the execution of patient instructions or consents [14,109]. In so doing, blockchain is widely portrayed as *solving* the tension between the need to respect the privacy and confidentiality of individual health records and recognizing the tremendous knowledge potential unleashed by pooling and aggregating patient data. Blockchain for health care scholarship has largely taken these claims at face value. Although various blockchain-enabled EHR projects are currently being experimented with, the preponderance of activity appears to reflect the views of industry experts, who advise that blockchain applications focusing on patient care records are best avoided in the early stages of the sector’s engagement with the technology because of the demanding and complex legal and regulatory requirements that apply to personal health information. Nevertheless, many believe that these demands can eventually be overcome, so that patient-controlled, blockchain-enabled EHRs will eventually emerge [91,117]. Even if the difficult and complex challenges and tensions identified in the preceding section can be satisfactorily overcome, whether these systems will live up to their promise depends upon the validity of underlying assumptions about their benefits, particularly in clinical contexts.

Academic literature concerned with health IT implementation has hitherto been dominated by the *technology acceptance model* (TAM), which originates from the perspective of health information systems research that studies the systems that support health and medical work [156]. This approach, developed jointly by doctors with an interest in computers and computer scientists with an interest in medicine, adopts a positivist outlook that regards the relationship between technology and their expected benefit as linear and causal [81]. Similar assumptions appear to underpin 2 distinct, albeit related, elements of the vision of blockchain-enabled EHRs offered by their proponents. The first assumption is that blockchain will enable the seamless integration of patient records currently stored in distinct *data silos,* thereby effectively providing patients and clinicians with a single, up-to-date, and comprehensive repository of each patient’s medical records over his or her lifetime. The second assumption is that configuring blockchain-enabled EHR systems to enable patients to control access to their records will ensure respect for privacy without impeding the transfer and sharing of patient data for clinical and other legitimate purposes.

In relation to the first assumption, Berg et al [157] highlighted studies of EHR implementation that emphasize the personalized nature of health care work, thus setting “natural limits to the possibilities of IT to revolutionise this work.” These studies suggest that the considerable energy and resources thus far devoted to various components of the EHR failed to substantially improve the quality or efficiency of frontline clinical work. Moreover, they argue that EHRs are *unlikely ever* to produce dramatic gains in the quality of care because of the importance of the creative human work needed to bridge the gap between clinical design and technical reality [50]. In contrast, biomedical literature often rests on a TAM model, evoking a belief that IT systems will make clinical information instantly available, implicitly assuming that *meaning* can be transmitted unproblematically along with the data contained in the EHR. This assumption also appears to underpin the *beautiful dream* of blockchain-enabled EHR systems, suggesting that, if properly designed, blockchain will enable more accurate, secure, and timely data access that will drive quality improvements across health care, including clinical care. However, studies from more sociologically informed traditions roundly reject this assumption, providing evidence that clinical data must be interpreted in context and *framed* before they become meaningful, and this is not achieved simply by placing information on an electronic platform that is accessible by multiple users [50]. These studies rest on assumptions that differ from those underpinning the TAM model (including understandings of what counts as *success* for the purposes of evaluating health IT systems), drawing on a wider range of methodological approaches [158]. They highlight the critical role of context, culture, and the values brought to bear by medical professionals in clinical environments, identifying the clinical consultation as a complex social encounter that occurs within a heavily institutionalized environment [50]. In particular, scholars from these traditions emphasize the role of values in clinicians’ understanding of what constitutes and how they seek to practice *excellent care* and the nature of clinical knowledge as *tacit, context-bound, and ephemeral rather than codifiable, transferable and enduring* [50]. These studies find that in many *failed* EHR projects, technical designers typically missed these subtleties and produced artifacts that fitted poorly with the situated nature of knowledge and the microdetail of clinical work practices [81].

Conversely, the TAM model assumes that the failure of EHRs to generate their expected benefits can be largely explained by shortcomings in EHR system design and implementation, resulting in user *resistance* to the technology [50], which can be overcome by system design improvements and more sensitive implementation. One of the most significant lessons from studies of EHR adoption is that considerable clinician resistance to these systems has often resulted from the unintended but radical alteration and disruption of clinical workflows and patient interactions accompanying the introduction of EHRs, adversely affecting physician workload [81,122,159]. In addition, the data captured in these records have been described by a legal expert as often error prone, incomplete, unprotected, and dispersed across numerous organizations unknown to the patient [130]. However, if used as *middleware* to support the integration of EHRs across disparate data silos, blockchain technologies should not, in theory, affect clinical workflows or practices, as they operate as a *backend* technology. In other words, blockchain should not exacerbate workload burdens or introduce additional integration work of the kind that has typically accompanied EHR implementation. However, HIMSS [160] warns that:

Blockchain technology is meant to operate on the back end and should avoid, where possible, adding additional steps for the end user. If this technology is poorly implemented without a strong federated approach, workflow may be negatively impacted, potentially affecting the quality of care.

Adding a blockchain-enabled solution without considering its applicability to a use case, potential value, or relationship with a partnering stakeholder, could add a layer of complexity to an already convoluted health IT ecosystem. Furthermore, if this complexity detracts from the end goal of sound care, ethical and other questions may arise from the implementation of this kind of solution that is not well suited for the coordination and delivery of care.

This cautious view, offered by one of the health care IT sectors’ leading industry bodies, suggests that one cannot assume that implementation of blockchain as a *backend* technology will not add further system complexity simply by avoiding any change to the *front end* experience of users [98].

The second assumption underpinning the promise of blockchain-enabled EHR systems—that they will enable patient data sovereignty and empowerment by providing patients with fine-grained control over who may securely access their health care records and on what terms—resonates with broader societal shifts in favor of *self-service economy* models across many sectors [161]. Blockchain for health care advocates assumes that patient-controlled health care records will resolve dilemmas arising from the tension between ensuring compliance with privacy and confidentiality norms and duties and fostering the aggregation of patient care records for medical research and other legitimate purposes [162]. This assumption is likely to be both mistaken and naive for several reasons. First, although it is essential that patients are enabled to exercise their rights of informational self-determination in relation to health and care information that directly pertains to them [163], some patients will enthusiastically embrace the capacity to control access to their medical records [164], whereas others will not [165]. A significant proportion of patients (including children and those with mental illness or disability) cannot make informed decisions about access to and sharing of their clinical data; therefore, alternative consent models will be needed. Second, there will be circumstances in which patient consent cannot be secured; however, the ability to access that patient’s health records will be necessary, particularly in emergency situations. Third, in other circumstances, patient rights to data privacy and confidentiality may be legitimately overridden by compelling public interests, particularly when appropriate technical and organizational measures are taken to safeguard those interests so that patient consent is neither legally nor ethically needed to justify access to their records. For example, the public interest in data sharing and access can, particularly in times of public health care emergency, justifiably override individual rights to privacy and the need for patient consent to data sharing.

Taken together, the vision of *patient data sovereignty* underpinning blockchain-enabled health records exhibits a *techno-solutionist* mindset, reflecting a belief that technological fixes can be used to *solve* complex social problems [166]. Critiques of this kind draw attention to the complex and messy set of interacting norms, practices, and dynamics that arise in the real-world contexts for which simple technological *solutions* are unlikely to be found, which the disappointing experience of EHR implementation has vividly demonstrated. Despite this, the underlying *information as property* paradigm that informs these beliefs appears to hold considerable sway in contemporary debates about data governance, both within health care and beyond [167]. However, as the German Data Ethics Commission points out, supporting and promoting the practical capacity of patients to access their health care records and take an active role in decisions about access to those records does not necessitate a *propertisation* model of data governance, which underpins the views of those who favor the making of micropayments to data subjects in return for access to their personal data [163,168,169].

Apart from unfounded assumptions that technological access controls built into health IT systems can be expected to successfully mediate and resolve the complex interests in patient data, any such shift in favor of patient sovereignty over health care records may reflect an underlying reconfiguration of the ethical basis upon which patients receive care. Rather than characterizing patients as vulnerable individuals and beneficiaries of care provided by an expert clinician who is professionally obliged to act in their best interests, patients may increasingly be expected to *self-manage* their own medical records based on largely unexamined claims that this will *empower* them to exert greater control over their own care [85]. However, if these systems are configured in ways that force adherence to norms that are at odds with the basic cultural and moral norms of contemporary health care settings, this might provoke backlash. This might occur, for example, if systems are designed to shift responsibility for care to patients and away from clinicians and care providers in ways considered contrary to the professional duty and commitment of clinicians to act in the best interests of their patients [170]. Alternatively, if patients are unable to make meaningful decisions about the sharing of their data, they are likely to stick with defaults; however, these might fail to reflect legitimate patient expectations and existing informational privacy and confidentiality norms. These and other similar risks underline the importance of meaningful stakeholder engagement in the design and implementation of blockchain systems, as well as the importance of more critical engagement with the logic of *empowerment* that frequently accompanies their promised benefits [50].

Accordingly, the vision underpinning blockchain for health care’s *favorite use case*, which is capable of seamlessly integrating patient-controlled EHRs accessible by clinicians in real time wherever located, is based on problematic and unrealistic assumptions. Their promised benefits are therefore unlikely to be realized in practice. Even if blockchain technologies can operate as *middleware*, enabling organizations to access and retrieve patient health care records stored at different sites in real time, blockchain cannot ensure that the information stored in those records is an accurate representation of the underlying real-world events they purport to reflect. Conferring responsibility for making access decisions on patients themselves will not avoid conflicting and sometimes difficult trade-offs between respect for patient privacy and other rights and legitimate interests implicated in health care. In other words, the health care *data silo* problem is only one aspect of a much larger, more complex set of needs, rights, and interests associated with medical and health care information, which are likely to continue to defy hard-coded solutions.

However, one of the most valuable insights arising from *technology-in-practice* studies of EHRs for understanding the real value that blockchain-enabled EHRs might create arises from the recognition that there are two conflicting work processes in play with EHRs: immediate clinical care (primary use) on the one hand and tasks such as audit and research, which are one step removed from the clinical encounter (secondary use) on the other. These studies show that when EHRs are used as a formal tool (eg, with structured templates and a requirement for data to be coded), they often slow down and frustrate the clinical encounter but can greatly accelerate secondary uses of clinical data. Accordingly, Greenhalgh et al [50] argue that, rather than promising that the EHR will *save time* or *make clinical care more efficient*, a more honest message would be that creating accurate and complete clinical records requires the sacrifice of time and effort by frontline clinical and admin staff, which is (sometimes) justified by more benefits for efficient business processes (eg, billing) governance and research. In other words, the real value of blockchain-enabled health records might rest in their secondary use, enhancing health care administration and medical research rather than in substantially improving clinical care.

### Study Strengths and Limitations

This study has both empirical and theoretical strengths. Its strengths are 3-fold. First, by focusing attention on the health care sector’s experience in attempting to use blockchain technologies for specific purposes, it fills an important gap in the existing literature by offering a more grounded, evidence-based but theoretically informed appraisal of blockchain’s prospects for transforming health care, unlike the burgeoning academic literature that has focused overwhelmingly on the technology’s *potential* to solve health care problems. Second, it identifies and critically examines a series of complex, multidimensional implementation challenges that must be satisfactorily confronted if blockchain technologies are to be widely taken up within health care contexts, illuminating the nature and dimension of these difficulties by referring to a range of theoretical perspectives. Third, the critique offered here contrasts sharply with the considerable hype that accompanied the emergence of blockchain technologies. It provides a more theoretically and empirically grounded appraisal of the oft-proclaimed radically transformative potential of blockchain technologies in a specific domain, notably within complex health care environments that are typically saturated with technologies, many of which are highly sophisticated and often safety-critical.

The limitations of the study rest in the lack of publicly available, authoritative data concerning blockchain uptake in health care. Hence, the snapshot provided by the study of current and ongoing industry engagement and experience with blockchain relies heavily on web-based investigations of gray literature, particularly industry publications, to investigate the focus and tenor of discussions about these technologies in health care and the trajectory of their development. This methodological approach was limited in several significant aspects, insofar as it relied on unconfirmed industry sources publicly available on English-language websites as a substantial source of evidence for understanding the current state of blockchain development and implementation in health care contexts. As the accuracy of claims made on public websites is practically impossible to verify and often fails to provide significant financial information about the level of investment or revenue generated (or expected to be generated), the findings on blockchain take-up in the health care sector are not comprehensive, particularly given the growing interest in developing blockchain applications in non–English-speaking countries (eg, China [171,172] and Japan [173]).

Although significant assurance about the reliability and robustness of the study’s provisional findings was obtained by eliciting feedback from industry experts through the second focus group workshop while cross-checking the validity of these provisional findings against the published insights of industry specialists [174-176], the findings concerning the state of industry experimentation and maturity offered here are neither authoritative nor comprehensive. Nevertheless, they provide worthwhile insights into the general state of the health care sector’s engagement with blockchain technologies to date, the particular domains and applications in which this engagement is occurring, and the current direction of travel. Accordingly, these findings offer a useful reference point for critical reflection in the context of future investigations of blockchain in health care and beyond.

### Conclusions

This paper has demonstrated that the health care sector’s engagement with blockchain technology has grown steadily since 2016, with the most promising activity occurring at the intra- and interorganizational level to enable data sharing and cooperation in health care administration and medical research. Although various blockchain initiatives have appeared in response to the COVID-19 pandemic [177], in which the rapid spread of misinformation intensified the importance of ensuring trustworthy health care information management, industry experts suggest that they have not hitherto taken root [178]. Contrary to the focus of the academic literature, the health care sector’s engagement with blockchain has not focused primarily on enabling and integrating patient-controlled EHRs into clinical practice. It is in nonclinical contexts that early-stage health care engagement with blockchain is now producing evidence that blockchain-enabled cooperation can create both collective and individual value for network members that is greater than the sum of its parts, generating potentially significant efficiency gains [121]. Accordingly, blockchain in health care cannot currently be dismissed as a passing fad. Although scholars of innovation studies have identified various *barriers to innovation* that arise when technological inventions seek to cross the *valley of death* during the development phase of innovation, this literature has tended to treat technological inventions as a *black box*. In contrast, the analysis offered here highlights the importance of attending to the design and functionality of the technology itself and the particular health care contexts and fields of practice into which it might be applied in ways that may deepen and enrich innovation studies literature that has tended toward a high level of generality and abstraction.

This study identified 6 complex and multidimensional challenges and 3 normative tensions that must be resolved if blockchain technologies are to be made readily implementable in health care. Negotiating and overcoming these challenges and tensions will require considerable commitment, including time, investment, and willingness by health care organizations to take substantial risks, experiment, learn, and share knowledge across the sector. It suggests that the technology’s progress through the development process will be difficult and lengthy. Moreover, as the National Advisory Group on Health Information Technology advised in its 2016 report, “implementing health IT is one of the most complex adaptive changes in the history of healthcare, and perhaps of any industry” [179]. Another leading expert observes that “while policy makers are calling for technology to be implemented rapidly and at scale, the reality is that when dealing with the multiple complexities of health and care, it is extremely difficult to go beyond small-scale demonstration projects” [85].

Even if blockchain succeeds in crossing the valley of death, it does not necessarily follow that these technologies will live up to their promise. The underwhelming experience of health IT offers a sobering reminder, with Kellermann and Jones [180] noting that despite the increased use of health IT in the United States, with aggregate annual national expenditure on health care growing from US $2 trillion in 2005 to roughly US $2.8 trillion in 2013, the efficiency of patient care is only marginally better. In the United Kingdom, 10 years after the launch of the National Program for IT, which promised to revolutionize care in the English NHS [181,182], it was described by the UK Secretary of State for Health as a “huge disaster that became impossible to deliver” despite costing £3.66 billion (US $4.84 billion) [183,184]. These experiences highlight the serious gap between the grand visions of IT’s transformative potential in health care and the fraught nature of implementation, given the inherently complex, dynamic, and context-driven nature of health care. They throw into high relief the inherent difficulties associated with seeking to *hard code* norms into complex sociotechnical systems in ways that that can satisfactorily resolve and mediate a wide variety of often conflicting rights, interests, expectations, norms, and risks associated with information sharing in dynamic, complex but safety-critical health care settings.

Although the *blockchain for health care promise* is rooted in a belief that blockchain systems will facilitate secure data sharing while enabling more fine-grained management of health care data, thereby creating *more and better data* that will drive improvements across the sector, real-world engagements and experiences with the technology in health care settings to date suggests that blockchain’s core *value proposition* is more nuanced and narrowly framed. Blockchain’s value generally (both within health care and beyond) rests primarily on its capacity to provide an automated, mathematically verifiable, highly secure, and therefore, highly trustworthy record-keeping function. It is this functional capacity that supports the technology’s nascent potential as *middleware*, facilitating various kinds of health care data sharing and exchange, particularly between health care organizations where a lack of social trust rooted in institutional incentives for opportunistic behavior might be replaced with the computational trust that permissioned blockchain systems can provide. In so doing, blockchain systems may enable new forms of cooperation that generate shared value from the pooling of health care data, although whether these networks can develop governance strategies that can successfully and sustainably mediate the relevant interests of network participants remains to be seen.

However, the difficulties of ensuring that the ledger accurately, systematically, and comprehensively tracks and reflects the real-world phenomenon that it claims to record may mean that the envisaged benefits of blockchain’s automated *track and trace* function may not be fully realized. Computer scientists and mathematicians have long recognized the *garbage in, garbage out* problem: the quality of output is determined by the quality of the input. Blockchain technology cannot guarantee that data arriving at and recorded in the ledger is an accurate, complete, and comprehensive record of the underlying real-world events that it purports to track; however, blockchain advocates typically overlook this critical vulnerability. In other words, although blockchain systems can deliver valuable functionality in terms of automatically and securely recording the handling of data items (for example, when a digital record has been viewed by a particular user), they cannot guarantee the integrity of the underlying data.

Nevertheless, blockchain’s automated audit function might offer real and significant value because, by creating a highly secure and verifiable record, it offers a vehicle through which organizations can *demonstrate compliance with data handling norms* at scale, thus operating as a form of *RegTech*. In this way, blockchain as middleware can automatically and securely track and record the location and handling of data items. Therefore, it might have real potential to facilitate the *integration* of health data *silos* stored in a variety of locations across multiple organizations using different IT systems [185]. The value of this contribution could potentially be significant, reducing costs and improving efficiency, transparency, and security, and could be widely applied across any sector in which sensitive data generation, processing, and sharing occur in large volumes.

Blockchain is frequently portrayed as offering the potential to deliver radical and positive transformations to many sectors, including health care. This study has identified significant activity within the health care sector aimed at exploring how blockchain technologies might deliver real value. It has been found that the technology is still in its infancy and that the nature and scale of its value to health care remain unproven [29]. Experience of early health care sector engagement has shown that the relevant question is not *how can blockchain be used* in health care settings but *under what conditions can blockchain be realistically expected to provide a viable, scalable and efficient solution to a concrete health care problem in real-world health care settings that would add significant value relative to alternative solutions?* In other words, when considering whether to engage with blockchain technologies, organizations must ask themselves whether a blockchain is really necessary to solve the problem at hand or whether other alternative approaches are preferable.

This shift in mindset is reflected in the divergence between the *rhetoric* of blockchain-enabled health care data sharing, which focuses primarily on the capacity of blockchain technologies to enable the seamless yet secure sharing of patient records that will enhance clinical decision-making, and the *reality of* engagement with blockchain, which has largely avoided clinical contexts. Instead, the most innovative health care blockchain applications have occurred at the interorganizational level, enabling the sharing of administrative data between health care organizations to improve the efficiency of health care administration. The most recent initiatives are aimed at facilitating shared access to the secondary use of patient data for medical research through cooperative endeavors to train algorithmic models on data sets in a secure, privacy-preserving manner.

However, to succeed in this endeavor, there remain very significant challenges associated with achieving social trust and cooperation, including the need for the effective and legitimate governance of blockchain networks, that no amount of technological sophistication or hard-coded solutions can, on their own, satisfactorily resolve. It remains to be seen whether these and other tensions inherent in these incipient forms of blockchain-enabled *coopetition* between health care organizations will be satisfactorily overcome and whether blockchain applications in health care might be characterized as *radical* or *incremental* technologies. Accordingly, the possibility of blockchain technology precipitating a *revolution* in the delivery of health care of the kind envisaged by blockchain for health care advocates appears to have been exaggerated. Nevertheless, there are real prospects of using blockchain in ways that could lead to genuine health care improvements. However, progress toward this goal is likely to occur slowly, incrementally, and more *by stealth* rather than through radical and rapid *disruption* over a short period and—at least in the first instance—likely to be concentrated across a much narrower range of applications than the blockchain for health care promise suggests.

## Appendix

Multimedia Appendix 1 Summary of systematic literature reviews of blockchain in health care.

Multimedia Appendix 2 Summary of method and findings mapping blockchain in health care by Motsi-Omoijiade and Kharlamov [21].

Multimedia Appendix 3 Focus group participants and proceedings.

## Abbreviations

B2B: business-to-business
EHR: electronic health record
HIMSS: Healthcare Information Management and Systems Society
IT: information technology
NHS: National Health Service
ProCredEx: Professionals Credentials Exchange
TAM: technology acceptance model
