# Supplementary material for: The Health Care Sector’s Experience of Blockchain: A Cross-disciplinary Investigation of Its Real Transformative Potential
Source: J Med Internet Res. 2021 Dec 20;23(12):e24109. doi: 10.2196/24109 (PMC8726042; doi:10.2196/24109)
Supplement: Multimedia Appendix 1 [file jmir_v23i12e24109_app1.docx]

## Multimedia Appendix 1:

## Summary of systematic literature reviews of blockchain in healthcare

| **Citation** | **Databases searched & date last updated** | **No of papers reviewed** | **Findings concerning implementation, take-up and maturity** |
| --- | --- | --- | --- |
| **Hölbl M, Kompara M, Kamišalić A, Nemec Zlatolas L**. A systematic review of the use of blockchain in healthcare. Symmetry 2018; 10(10):470. [doi.org/10.3390/sym10100470](https://doi.org/10.3390/sym10100470) | June 2018  Google Scholar  Web of Science  IEEE Xplore,  PubMed  Medline EBSCO  Springer-SpringerLink  Elsevier ScienceDirect  ACM Digital Library  Cochrane Central | Total: 33  Comprised of 14 conference papers,  11 journal papers,  3 patents & 5 grey literature items | Of the 33 selected papers, only 5 presented an implemented and deployed solution. In most publications, proposals are made that could be implemented, but mostly were not. Most of the publications use blockchain for data sharing, health records and access control. Blockchain is rarely used for supply chain management, audit trail management and other scenarios, like drug prescription management and auditing. Most publications are “highly theoretical” and “do not include a prototype or a real-life implementation.” |
| **Agbo C. C. Mahmoud Qusay H. Eklund J. M.** Blockchain Technology in Healthcare: A Systematic Review. Healthcare 2019;7(2): 56. <https://doi.org/10.3390/healthcare7020056> | December 2018  PubMed  IEEE Xplore  Web of Science Scopus | 65 | Of the 65 papers, 37 (57%) were written by authors in academic institutions, while only 9% (6 papers) were from the industry. The remaining 34% (22 papers) were products of collaborations between industries and the academia. “What this shows is that since this technology is still maturing, industrial players may still be reluctant to start adopting the technology into their operation.” The majority of the papers were published by authors in China 26% (17 papers) and USA 23% (15 papers) of which 45 (69%) are technical papers and 19 (29%) are reports (defined as publications that lack a technical contribution). A substantial portion 48% (31 papers) concentrate on the application of blockchain in the management of electronic medical records. “In general, the technology is still maturing and even when prototype applications are developed, in some cases, they are just for experimental purposes with very basic functionalities. But there are papers that present implementation details of applications that have been developed for the various use cases. Our study shows that blockchain has many healthcare use cases including the management of electronic medical records, drugs and pharmaceutical supply chain management, biomedical research and education, remote patient monitoring, health data analytics, among others. More research is needed to better understand, characterize and evaluate the utility of blockchain technology in healthcare, and to supplement ongoing efforts to address the challenges of scalability, latency, interoperability, security and privacy for these uses.” |
| **Hasselgren A, Kralevska K, Gligoroski D, Pedersen SA, Faxvaag A**. Blockchain in healthcare and health sciences—A scoping review. International Journal of Medical Informatics 2020;134. DOI: [10.1016/j.ijmedinf.2019.104040](https://doi.org/10.1016/j.ijmedinf.2019.104040) | 10 Oct 2018  MEDLINE  Embase  Cochrane Library  Scopus  Google Scholar  Compendex  Inspec  ACM  IEEE | 39 | All of the 39 selected papers included proof of concept study design. Of these, 11 offer a hybrid of proof of concept design and a case study design. Most studies were associated with Chinese research groups (42% - 16 papers) followed by groups in the USA (20% - 8 papers). The frontier of research, as portrayed in this review, show that blockchain based solutions are currently being explored in a few EHR, PHR and clinical trial system use cases. Research on the use of blockchain in healthcare is now established as an academic field, and the number of publications are increasingly rapidly. |
| **Durneva P, Cousins K, Chen M.** The Current State of Research, Challenges, and Future Research Directions of Blockchain Technology in Patient Care: Systematic Review. J Med Internet Res 2020;22(7):e18619. DOI: [10.2196/18619](https://doi.org/10.2196/18619) | 1 December 2019  Cumulative Index of Nursing and Allied Health Literature (CINAHL)  PubMed  Embase  Web of Science | 70 | Examines current state of research on blockchain technologies in patient care only, excluding all other healthcare applications. The selected papers were classified into 3 groups representing the main health IT (‘HIT’) challenges that blockchain can address: (1) data security and privacy, (2) interoperability, and (3) health care quality outcomes. Most of the research reviewed focused on blockchain’s use to strengthen HIT security or patients’ privacy during health data exchange or access. With 51% of the research focused on medical information systems such as electronic health record and electronic medical record, and 53% of the research focused on data security and privacy issues, this review shows that HIT research is primarily focused on the use of blockchain technologies to address the current challenges HIT faces. Although Blockchain presents significant potential for disrupting health care, most ideas are in their infancy. |
