# Supplementary material for: The Health Care Sector’s Experience of Blockchain: A Cross-disciplinary Investigation of Its Real Transformative Potential
Source: J Med Internet Res. 2021 Dec 20;23(12):e24109. doi: 10.2196/24109 (PMC8726042; doi:10.2196/24109)
Supplement: Multimedia Appendix 2 [file jmir_v23i12e24109_app2.docx]

## Multimedia Appendix 2

## Summary of method and findings mapping blockchain in healthcare by Motsi-Omoijiade and Kharlamov (2021)

The investigation by Motsi-Omoijiade and Kharlamov (see I Motsi-Omoijiade and A Kharlamov 'Blockchain for Healthcare Applications and Usecases' in Pollicino O and De Gregorio G (eds) *Blockchain and Public Law: Global Challenges in the Era of Decentralisation* ( Edward Elgar 2021, 136-189) ISBN: 978 1 83910 078 9) sought (among other things) to identify and map evidence of real-world engagement with blockchain technologies in the healthcare sector. Each blockchain application identified was classified by reference to its primary function, level of maturity and the primary geographic region from which the relevant firm providing the blockchain application is headquartered or registered. The primary data sources consulted for that study consisted of publicly accessible researcher-compiled blockchain for healthcare databases published in the English language, including those compiled by the Centre for Biomedical Blockchain Research^^[[1]](#footnote-1)^^, Caravos^^[[2]](#footnote-2)^^, Kuraitis^^[[3]](#footnote-3)^^, Miller^^[[4]](#footnote-4)^^, Stoffregen^^[[5]](#footnote-5)^^ and Mire^^[[6]](#footnote-6)^^. These databases were consolidated, cross-checked, updated and triangulated with each other, and with results from publicly available sources, including available grey literature. This resulted in a list comprised of 128 industry-sourced blockchain for healthcare applications at the study cut-off data of 30 November 2019. Excluded from this list were blockchain applications without a specific focus on healthcare, recognized scams, and failed, discontinued or unsubstantiated blockchain for healthcare Initial Coin Offerings (ICO’s). Salient findings are summarised below, with permission from the authors:

**(1) Blockchain for healthcare applications’ by function**

A blockchain for healthcare ‘application’ was defined as a proposal for utilising blockchain to provide a specific functional healthcare purpose. Accordingly, single blockchain system identified from the website review might provide multiple applications. As at 30 November 2019, that study had identified **128 healthcare blockchain applications**. This were classified based on their intended application and function. The study found that of these:

- a substantial minority, 50 out of 128 (39%) focused on applications to enable user-control over various forms of personal health data which can be securely shared via a blockchain platform;
- a significant proportion, 34 out of 128 (27%) were primarily concerned to facilitate healthcare administration;
- a similarly sized proportion, 31 out of 128 (24%) were intended to support patient care and care management (often to facilitate remote care consultations between patient and clinician located elsewhere), to encourage healthy behaviours and to help prevent disease at the population level; and
- a small proportion, 13 out of 128 (10%) were primarily concerned with facilitating data management for medical research.

(a) **Patient care support, management and disease prevention**: these applications refer to a variety of services offered either to users directly (including services aimed at helping individual patients achieve their health goals particularly through attempts to incentivise healthy behaviours) or for health professionals to assist in the provision of various forms of care support to patients (including applications to help coordinate patient care, to facilitate remote care, to facilitate the achievement of public health goals). These accounted for 31 out of 128 (24%) of the total number of blockchain for healthcare applications identified.

(b) **Data management for** **medical research**: included those aimed at facilitating secure data management necessary for the development of new medicines or medical procedures or to improve those already available. These category formed only 12 out of the 128 (10%) of the total surveyed blockchain for healthcare applications, the smallest proportion of applications surveyed. This category included applications for clinical trial management, research data management and administrative purposes and to facilitate secure research data sharing. Relative to the other categories of applications, blockchain applications to facilitate the management of data for medical research is relatively mature, with the majority of surveyed applications (8 out of 13) being available for use.

(c) **User control over electronic health data**: these applications were designed to enable individuals to exercise control over specific kinds of personal health data, ranging from ‘wellness’ data collected from health and wellness wearables through to individual medical care records. This category represented the most popular use of blockchain in healthcare with 50 out of 128 (39%) of all surveyed applications. These were concerned with facilitating and enabling individuals to share their own health data with third parties either for financial remuneration, to facilitate data donation for research purposes or for identifying and seeking alternative care options.

**(d) Healthcare administration:** refers to the oversight and management of the healthcare system in order to ensure the coordinated delivery of healthcare and the efficient management of medical facilities. The healthcare administration-related applications identified in the survey cover the areas of (i) billings, insurance, payments and contracting (ii) supply chain and provenance including of pharmaceuticals and medical credentials (iii) in-hospital permissions and consent management. Healthcare administration formed 34 out of 128 (27%) of all surveyed blockchain for healthcare applications, which is the second largest group of applications after patient control of patient and health records. The majority of these are in development.

**(3) Level of maturity**

Each application was further classified by its level of maturity as either

- ‘available’ (ie those with a blockchain for healthcare product, application, platform or service that is live and readily accessible through the company’s or a third party’s website. These firms had typically produced mobile applications (apps) that could be downloaded from platforms such as Google Play or the Apple App store and/or lists of verified partners and clients); or
- ‘developing’ (ie where no product or service has yet been brought to market).

Aside from e-Estonia, the website review found that active engagement with blockchain technology in healthcare began in or around 2016, primarily in the USA and, to a lesser extent, in Europe, with several recent initiatives occurring at the transnational level. Yet less than half (60 out of 128) of these had a commercially available blockchain product or service, with most (69 out of 128) at the experimental or development stage at the survey cut-off date. Against this background, e-Estonia, the Estonian government’s national health record management system is unique. It is a health information system management platform built on a publicly-owned blockchain to assure the integrity of retrieved electronic medical records while also creating system access logs^[[7]](#footnote-7)^ to facilitate efficient, secure and traceable health administration tasks. It is designed to enable the Estonian national health ministry to compile health data statistics at the national level to facilitate the measurement of health trends, track epidemics, and help facilitate the efficient utilisation of health resources.

**(4) Geographic location**

Of these applications, 64 of 128 applications (50%) were geographically headquartered or incorporated in the USA with the UK as the next-most popular site of activity comprising 12 of 128 applications (9%).

**Blockchain for healthcare applications by category**

**Table 1: Patient care support, wellness, public health**

|  |  |  |  | **Patient care and disease prevention** |  |
| --- | --- | --- | --- | --- | --- |
|  | **App/Platform** | **Firm** | **Maturity** | **Description** | **Location** |
|  | Hayver | Hayver | Available | Blockchain monitoring platform to address drug and alcohol addiction using a flexible open architecture and cryptocurrency incentives | USA |
|  | MyPCR | Guardtime | Available | MyPCR provides patients with instant access to their primary care info, personal care pathways and medication adherence support via their smartphone | Switzerland |
|  | Healthcoin | Healthcoin | Available | An application that monitors diabetes related biomarkers and issues tokens for measurable improvements to health | USA |
|  | Eterly | Eterly | Available | Health and fitness advisor app focusing on longevity using token-based incentives | Canada |
|  | WELL | WELL | Available | A blockchain app that monitors data from wearables to incentivise and monetise healthy choices | USA |
|  | Humanitiv | Citizen Health | Available | An operating system for health & wellness that helps motivate and incentivise users to pursue healthy lifestyles with the aid of wearable tech | USA |
|  | SciCHAIN | SciCoins | Developing | A shared universal ledger designed to track patient data and monitor health and wellness using data from wearables | USA |
|  | LifesDNA | LifesDNA | Developing | Blockchain AI search engine and marketplace for wearables, healthcare and lifestyle info | UK |
|  | Amchart | Amysys | Developing | A patient-driven EMR on a public-private blockchain with AI for analytics and incentive-driven models to promote better outcomes | USA |
|  | AIM | Aimedis | Developing | Incentivised healthcare through a medical ecosystem and a social medical network | Netherlands |
|  | Gainfit | Gainfy | Developing | An incentive platform for fitness and healthcare | USA |
|  | Clinicoin | Clinicoin | Developing | An open source wellness platform that rewards users with cryptocurrency for engaging in healthy behaviours | USA |
|  | Coinhealth | Coinhealth | Developing | A health-focused platform that rewards users with Coinhealth tokens for healthy behaviours and allows users to share data with chosen providers | USA |
|  | Nano | nanoVision | Developing | Makes wellness recommendations by connecting users with health resources and experts | USA |
|  | Curaserve | Curaizon | Developing | Platform for improving drug adherence through big data delivered via blockchain that allows collection of fully anonymised data concerning when and how patient takes medication | UK |
|  | Dentacoin | Dentacoin | Developing | A dental care ecosystem that promotes preventive dental behaviours by motivating users to improve their oral hygiene habits | Netherlands |
|  |  |  |  | **Care coordination to assist direct care provision** |  |
|  | Etheal | Etheal | Available | Blockchain-based global directory of health professionals to help patients search for, and book, health specialists from around the world, helping them save on elective care costs | Estonia |
|  | Bowhead Health | Bowhead Health | Available | Offers a platform comprised of a hardware device that monitors a user’s biometric data to dispense personalised medicine and supplements | Singapore |
|  | Aidoc | Aidoc | Available | Combines blockchain and AI to provide real-time monitoring of data that can detect abnormal health status and provide advice accordingly | China |
|  | Panacea | RoboMed Network | Available | A medical platform managed by a blockchain token, designed to provide remote medical care | Russia |
|  | Rejuvan | BitMED | Available | A blockchain and AI telehealth platform that offers users on-demand 24/7 remote access to medical practitioners via text and video | USA |
|  | DocHealth | Doc.com | Available | A blockchain platform that offers ‘free’ remote care in exchange for user medical data | Mexico |
|  | Medvice | Medvice | Available | A medical consultation platform using AI and blockchain technology to facilitate remote clinical consultations | Netherlands |
|  | Alphacon | Alphacon | Evaluation | A blockchain platform that analyses genes, functional medicine, immunity and smart devices to provide remote healthcare optimised for each user | Singapore |
|  | Minthealth | Minthealth | Developing | A global decentralised health platform that aligns healthcare stakeholders around the shared goal of improved clinical outcomes | USA |
|  | HealPoint | HealPoint | Developing | A blockchain-based AI powered healthcare platform that delivers a second opinion from multiple experts to reduce the risk of misdiagnosis | UK |
|  | Medicohealth | Medicohealth | Developing | Telemedicine platform that allows patients to share data with doctors | Switzerland |
|  | Yazom Care | Yazom | Developing | Blockchain platform aimed at enabling users to access medical assistance from doctors and pharmacies regardless of location | Jamaica |
|  | Neo Blockchain | Neohealth | Developing | A medical information resource, clinical decision support tool and complete medical ecosystem using Neo blockchain technology | Malta |
|  |  |  |  | **Public health and disease prevention** |  |
|  | E-Estonia | E-Estonia | Available | National blockchain-based health record management system to assure the integrity of retrieved EHRs, creating system access logs to facilitate national data aggregation to measure and monitor health trends, track epidemics, and consumption of health resources | Estonia |
|  | Kinect Hub | Kinect Hub | Developing | Blockchain data-sharing platform to facilitate sharing of health outcome data and funds transfer facility for developing countries | UK |

**Table 2: Medical Research data management**

|  | **App/Platform** | **Firm** | **Maturity** | **Description** | **Location** |
| --- | --- | --- | --- | --- | --- |
|  | ARNA Panacea | ARNA Panacea | Available | Blockchain based platform to enable processing of clinical trial data and administrative support for researchers during R&D phase | Russia |
|  | Curecoin | Curecoin | Available | Cryptographic utility token dedicated to replacing ASIC mining with protein folding computations to help scientists find new medicines in lieu of mining hashes | USA |
|  | Shivom | Shivom | Available | Blockchain based platform utilising smart contracts to verify ownership of DNA data and authorise genomic data transactions between consumers, enterprise and organisations | Germany |
|  | Lifeledger | ConsliX | Available | A blockchain-based clinical trial platform to address the imperatives and incentives in clinical trial management | India |
|  | DNATix | DNATix | Available | An accessible, secure, anonymous platform for genetic labs, healthcare providers and genetic researchers working with large volumes of genetic data | USA |
|  | Bitmark | Bitmark | Available | App used to track health data and consent for medical research. Pfizer using Bitmark for clinical trial matching at cost effective scale | Taiwan |
|  | FarmaTrust | FarmaTrust | Available | Blockchain and AI-based provenance systems for clinical trials, medical devices, cell and gene therapy and pharmaceutical tracking | UK |
|  | Longensis | Longenisis | Available | Blockchain based consent management service for doctors and patients that streamlines data collection for medical research | Hong Kong |
|  | Blockchain Health | Blockchain Health | Developing | Blockchain platform that provides advanced auditing for medical research assuring that the chain of custody for sensitive health data is readily auditable | USA |
|  | Translo | Translo | Developing | A blockchain-based platform for sharing biomedical data in a decentralised, secure, efficient and permissioned manner | USA |
|  | Digital Sample Manager | H Blox | Developing | Uses DLT for sample tracking that ensures a streamlined, secure, transparent process across participants providing real time status updates that can be tracked efficiently, eliminating lost sample problems. | USA |
|  | BlockRx | BlockRx | Deveolping | A blockchain platform that provides a comprehensive solution to fully integrate life science researchers, biopharma, medical device manufacturers and healthcare providers to ensure pharmaceutical supply chains | USA |
|  | ADLT | iSolve | Developing | Enterprise blockchain solution for Biopharma Healthcare, Medical Device manufacturers and the life sciences, connecting manufacturers to patients and the patient to the rest of the healthcare system | USA |

**Table 3: Applications to facilitate user-control and access to their electronic health information**

|  | **App/Platform** | **Firm** | **Maturity** | **Description** | **Location** |
| --- | --- | --- | --- | --- | --- |
|  | Beat | Beat | Available | Blockchain platform that allows users to share data with sport sponsor, talent scouts, doctors, researchers and insurance companies | Germany |
|  | Bolt Protocol | Bolt Coin | Available | Sport and fitness ecosystem based on user generated data that can be shared and sold by users | Estonia |
|  | Emrify | Emrify | Available | App allows users to sync, view and share all their health records on a mobile device via a secure interface | USA |
|  | Health Wizz | Health Wizz | Available | Mobile platform to aggregate, organise and share medical records on the user’s terms | USA |
|  | HIT Platform | HIT Platform | Available | A blockchain based platform to support consensual sharing of health info between users and trustworthy organisations that give users a fair reward in return for data | Switzerland |
|  | Lympo | Lympo | Available | User controlled wellness and fitness data which can be exchanged via LYM utility tokens | Lithuania |
|  | Patientory | Patientory | Available | Application empowering users globally with a secure platform to transform and manage their health data to achieve actionable insights for improved health outcomes and well being | USA |
|  | Timihealth | Timi | Available | A web app that safely and anonymously stores your DNA genetic data and allows users to sell their data to other firms in the TimiHealth App | USA |
|  | Lifegraph | BurstIQ | Available | Personalised data integration across health records, wearables and social group | USA |
|  | Patient Truth | Embleema | Available | A tamper-proof holistic, longtitudinal and patient-centric worldwide health record interfacing with patients, physicians, care centers, health authorities and the pharmaceutical industry | ISA |
|  | Bramble | Hashed Health | Available | Data market place for trading digital representations of a healthcare service made available to purchasers to buy directly from providers thereby cutting out the need for an intermediary insurance company. | USA |
|  | CoverUs | CoverUs | Available | An application that allows patients to broker and sell their health data to interested parties | USA |
|  | Myclinic.com | Medical Chain | Available | Aims to put patient in control of their medical data, empowering them to share the single most comprehensive version of their record with every organisation in their medical network | UK |
|  | Universal Patient Index | Kalibrate Blockchain | Available | Blockchain platform that resolves patient identities across health information systems integrated via a FormDrop app allowing users to fill out any provider form anywhere | USA |
|  | #My.31 | Hu-manity.co | Available | Allows users to sell their data to Big pharma directly, cutting out the middleman | USA |
|  | Coral Health | Coral Health | Available | Blockchain platform that allows users to securely retrieve health records from places where they have received care and share conveniently via mobile device | Canada |
|  | Zenome | Zenome | Available | Plans to build a decentralised storage system for genomic data provided by network participants to facilitate sale to interested third parties | Russia |
|  | Nebula | Nebula Genomics | Available | P2P network for buying and selling genomic data using cryptographic techniques to secure data and ensure transparency | USA |
|  | LunaDNA | LunaDNA | Available | Community owned database that rewards individuals Luna Coins for contributing their DNA and other medical information | USA |
|  | GeneChain | Encrypgen | Available | De-identifies raw DNA data files allowing DNA buyers to search Gene-Chain profiles suitable for their projects and purchase de-identified genomic data with DNA tokens | USA |
|  | Doc.ai | Doc.ai | Available | A blockchain platform that enables users to earn financial compensation for collecting and allowing their encrypted and anonymised health data to be used in meaningful data trials | USA |
|  | CarePay | CareX | Available | Creates a secure digital payment platform providing transparent pricing of medical services that insurers can purchase and pay for directly while enabling patients to hold and anonymously share their health information | USA |
|  | Dovetail Lab | Dovetail Lab | Developing | Software allowing patient data to be shared to improve healthcare systems, healthcare products and services, as chosen by user | UK |
|  | eHealth First | eHealth First | Developing | A platform that will enable individuals to share at will complete personal health-related information, including clinical data, with stakeholders | Russia |
|  | HIE of One | HIE of One | Developing | Project to combine emerging standards for access authorisation and blockchain based self-sovereign identity into a patient-centred health record infrastructure | USA |
|  | MediChain | MediChain | Developing | A distributed ledger for patient’s medical data that allows patients to store their own data in a secure way and provides access to specialists anywhere regardless of the payer network or EMR | USA |
|  | MyMedis | MyMedis | Developing | Based on decentralised network technology,, the system uses an encrypted, distributed storage solution and offers easy access to medical data, providing ownership of medical records and health data | USA |
|  | Patient Directed | Patient Directed | Developing | A distributed data network that allows patients to have full control over access, utilisation and monetisaton of their personal health info | USA |
|  | Proof | Proof Work | Developing | Provides platform for self governance of health data, allowing others access via smart contracts using their portable management tool kit | USA |
|  | Stem Cell Innovations | Stem Cell Innovations | Developing | Will enable patients to connect with a Stem Cell Specialist instantly and in the same connection to decide when and how much confidential info to share with professionals | Gibraltar |
|  | Youbase | Youbase | Developing | Individual-centric exchange to enable a world where individuals have complete control over and are empowered to own their life data | USA |
|  | MedREC | MedRec | Developing | Platform for patients to store, manage and share EHRs and allow researchers to receive anonymised medical data in return for sustaining the network | USA |
|  | Ingeniciel | Ingeniciel | Developing | A blockchain-based health platform, providing users with an easy way to store and manage their medical records via a secure decentralised app | France |
|  | Iryo | Iryo | Developing | Zero-knowledge encrypted health data and public blockchain access control allowing patients to securely share their medical history | Slovenia |
|  | SSOT Health | SSOT Health Block | Developing | A smart contract and blockchain-powered health information network that enables patients to own and control their medical history data | USA |
|  | Zealeum | Zealeum | Developing | Connects personal health wallets to apps and wearables. Users receive tokens as rewards when they achieve specific health goals, for purchase and sharing of their data | Canada |
|  | Akiri Switch | Akiri | Developing | Software defined network that users blockchain to create a secure routing protocol for sharing healthcare data | USA |
|  | SYRI | AzzardHealth | Developing | Patient owned health data exchange built on blockchain which aims to empower patients by democratising health records, pulling and combining health data from multiple sources | Pakistan |
|  | Medibloc | Medibloc | Developing | Decentralised healthcare info ecosystem built on a blockchain platform allowing the tracing and recording of all transactions relating to patient’s healthcare records | South Korea |
|  | MedBlox | MedBlox | Developing | Enables free flow of health info by allowing patients or health proxies to validate and actively manage their existing EHR | USA |
|  | Patient Sphere | Open Health Network | Developing | Uses blockchain and smart contracts to enable consumers to control and manage their health information | USA |
|  | Compass | Betterpath | Developing | Platform that helps patients track down all their health data from all its sources and build a complete medical record for them to control, enabling doctors to identify the most suitable treatments | USA |
|  | Health Nexus | Simply Vital Health | Developing | Marketplace and matchmaking service for individuals, healthcare providers and researchers | USA |
|  | Trusted Health | Trusted Health | Developing | Platform to connect patients and doctors of rare or life-threatening diseases | UK |
|  | Medoplex | CitizenHealth | Developing | Marketplace where buyers and sellers transact directly in free market economy without insurance intermediaries | USA |
|  | HumanScape | Human Scape | Developing | A blockchain-based community that collects and compiles individual patient’s health data in a very systematic way to increase opportunities for drug development and clinical participation | China |
|  | Enome | Enome | Developing | Blockchain platform that allows consumers to control the use and benefits of their health and genomic data | Australia |
|  | Blupass | Blupass | Developing | Blockchain platform that aims to put control in the consumers hands to facilitate data transfers between providers and carriers for EHRs | USA |
|  | Health Passport | Secure Health Chain | Developing | Blockchain based digital health record that will act as a single source of truth for medical data that gives security, privacy and patient control over their medical data | Australia |
|  | Alfia | Alfia | Developing | Web App prototype that gives individuals control over their PHR and securely share with healthcare providers upon request | Unknown |

**Table 4: Healthcare Administration**

|  | Decent | Decent | Available | Smart contracts for health insurance for the self-employed | USA |
| --- | --- | --- | --- | --- | --- |
|  | Reimburse | Digipharm | Available | Blockchain-based solution for performance-based contracting of pharmaceuticals, medical devices and wider healthcare services | Switzerland |
|  | Solve Care | Solve Care | Available | Blockchain platform for decentralised administration and payments of healthcare | Estonia |
|  | Blockverify | Blockverify | Available | Extending anti-counterfeit solutions from luxury valuables to medications and pharmaceuticals | UK |
|  | Intelligent Healthcare Network | Change Healthcare | Available | Change Healthcare develops a wide range of products focused on claims management, payments and data management in the healthcare sector using Hyperledger Fabric | USA |
|  | ScalaMed | ScalaMed | Available | A mobile prescription exchange system for patients, doctors and pharmacists to manage, prescribe as well as dispense prescription medications | Australia |
|  | MediLedger | MediLedger | Available | Permissioned blockchain for tracking and tracing regulations to improve the operation of the supply chain the pharmaceutical industry | USA |
|  | Procredex | Hashed Health | Available | A blockchain exchange providing healthcare organisations with access to verified credentials information about medical staff | USA |
|  | Intiva Health | Intiva Health | Available | Platform for healthcare professionals for credential management, networking and marketplace | USA |
|  | SafeInsure | SafeInsure | Available | A decentralised insurance marketplace bringing equitable price discovery and global access to insurance policy-shoppers worldwide | USA |
|  | Medishares | Medishares | Available | A blockchain-based decentralised mutual aid insurance marketplace | Singapore |
|  | Meditect | Meditect | Available | A blockchain platform built to trace and authenticate provenance of medicines in Africa | France |
|  | Healthverify Consent | Healthverify | Available | Cloud and blockchain-based platform that helps healthcare organisations manage and govern consumer data-use permissions and consent across internal systems and external suppliers | USA |
|  | phrOS | phrOS | Available | Healthcare blockchain operating system for data sharing in a hospital context | Taiwan |
|  | MTBC | MTBC | Available | Medical billing company that offers a blockchain-based integrated EHR and practice management solution | USA |
|  | Grapevine Worlds | Grapevine Worlds | Available | Network for global institutions to share health records in an effective, inexpensive, quick and standardised data exchange | Austria |
|  | Days.Exchange | Days.Exchange | Developing | Unit-linked insurance plans for healthcare, life insurance and wealth management markets | Switzerland |
|  | Curisium | Curisium | Developing | Platform to allow payers, providers and life science companies to engage with patient-value-based contracts | USA |
|  | CuraBlox | Hs Blox | Developing | Users smart contracts to make payment disbursement, reconciliation and reporting of metrics easy to track and efficient, reducing time wasted waiting for payments and coordination | USA |
|  | RevBlox | HsBlox | Developing | Uses blockchain to prospectively help providers reduce claims denials and secure payment of patient financial responsibilities for care | USA |
|  | Universal Health Coin | Universal Health Coin | Developing | A privacy-focused, token-based healthcare finance system that utilises blockchain to arbitrage, decentralise and provision payment of health services | USA |
|  | Veris | Veris Foundation | Developing | The Veris Platform uses smart contracts to bring together healthcare service providers, insurers and banks to authorise the provisioning and payment of health services | USA |
|  | ElCoin | ElCoin | Developing | Seeks to combine the manufacturing of medical and healthcare equipment to a blockchain medical platform | Latvia |
|  | MedXchange | MedXchange | Developing | A supply chain system for handling transactions, data transfer and storage and payments for medical devices and supplies | China |
|  | Spiritus | Spiritus | Developing | Blockchain-based medical device life cycle management platform to allow all involved parties to record evidence of service for critical assets and infrastructure | UK |
|  | Mexc | Mexc | Developing | Ecosystem for purchasing Emergency Medical Services (EMS) products and services | Singapore |
|  | Synthium Health | Synthium Health | Developing | A blockchain enabled healthcare technology platform that addresses supply chain participants in the healthcare industry by linking manufactures, GPOs, distributors and suppliers | USA |
|  | Ambrosus | Ambrosus | Developing | Quality control and assurance of pharmaceutical products | Switzerland |
|  | BlockMedx | BlockMedx | Developing | Electronic prescription drug supply chain management focused on opioids and other notoriously mismanaged drugs | USA |
|  | Healthchain | Healthchain | Developing | Software solutions that integrate with existing healthcare management systems and workflows to make outdated technologies and paper-based processes more efficient | Canada |
|  | Blockpharma | Crystalchain | Developing | Blockchain platform for drug traceability and anti-counterfeiting | France |
|  | Connecting Care | Simply Vital Health | Developing | Uses care coordination and financial forecasting to help providers in bundled payments get insight on what happens when patients leave hospital | USA |
|  | Skychain | Skychain | Developing | Blockchain infrastructure aimed to host, train and use AI to reduce medical errors in healthcare | Russia |
|  | Healthlinkages | Healthlinkages | Developing | A blockchain and AI software system for collecting and analysing health data, enabling operations to save time while allowing better analytics and regulatory compliance | USA |

1. This in an independently crowdsourced Centre for Biomedical Blockchain Research ‘Healthcare and Biomedical Blockchains’ (2019) <https://db.biomedicalblockchain.org/companies_detail> Accessed 20 October 2019. [↑](#footnote-ref-1)
2. A Coravos, ‘*Open-source landscape map for healthcare-related blockchains’* (2018) <https://github.com/acoravos/healthcare-blockchains> Accessed 30 May 2018. [↑](#footnote-ref-2)
3. See <https://docs.google.com/spreadsheets/d/1TANOZmuYtVhyn1C9PV6YLsOBlQNOEOBExB7u2_Kkork/edit#gid=0> last updated October 2018) [↑](#footnote-ref-3)
4. R Miller, ‘An Update on Healthcare ICO’s’ (*Medium* 24 February 2019) <https://medium.com/@bertcmiller/an-update-on-healthcare-icos-e7ae25cc85ff > Accessed 20 October 2019. [↑](#footnote-ref-4)
5. E Stoffregen, ‘Blockchain Healthcare Ecosystem in 2018’ (*Medium* 6 July 2018) <https://medium.com/@erikstoffregen/blockchain-healthcare-ecosystem-d21631024454> Accessed 20 October 2019. [↑](#footnote-ref-5)
6. S Mire, ’40 Startups using blockchain to transform healthcare’ (*Disruptor Daily* 14 December 2018) <https://www.disruptordaily.com/blockchain-market-map-healthcare/> Accessed 20 October 2019. [↑](#footnote-ref-6)
7. E-Estonia, ‘e-Health Records’ (*E-Estonia* 2019) <https://e-estonia.com/solutions/healthcare/> Accessed 26 October 2019. [↑](#footnote-ref-7)
