# Supplementary material for: The Health Care Sector’s Experience of Blockchain: A Cross-disciplinary Investigation of Its Real Transformative Potential
Source: J Med Internet Res. 2021 Dec 20;23(12):e24109. doi: 10.2196/24109 (PMC8726042; doi:10.2196/24109)
Supplement: Multimedia Appendix 3 [file jmir_v23i12e24109_app3.docx]

## Multimedia Appendix 3

## Focus Group Participants and Proceedings

**Focus Group 1**

The first workshop took place on 10-11 October 2018 at The University of Birmingham, UK.

**Academic-Stakeholder Workshop Materials:**

Workshop participants were provided with an information pack setting out the aim of the workshop discussion in the context of the Wellcome-funded research project, its research questions and overarching objectives. In particular, the welcome message from the project PI stated:

“This workshop has brought together a select group of key individuals involved in building blockchain applications for healthcare together with world-leading academics from several disciplines including law, computer science, ethics, and healthcare governance in order to understand the opportunities and challenges associated with developing and implementing blockchain technologies for healthcare. Our aim in hosting this workshop is to provide a forum in which industry and academic participants can discuss and share their understanding of the opportunities, risks and challenges in seeking to utilise Blockchain in a healthcare context, and to help establish a network of individuals and organisations with a shared interest in blockchain applications for healthcare.

The overarching aim of the project is to map the legal, ethical, technical and governance challenges pertaining to the regulation of healthcare through blockchain by identifying, mapping, and critically examining the implications for utilising Blockchain in this context. In so doing, this project will begin to explore whether, and under what conditions, these technologies might be developed whilst remaining faithful to important ethical, legal and constitutional values.

Discussion will take place under Chatham House Rule (identity of workshop participants disclosed, with individual comments recorded but without attribution) although participants are free to indicate if they would like particular comments to be made off-the-record.

Academic collaborators participating in today’s workshop will be staying overnight and will reconvene on Thursday morning to reflect on the workshop discussion. In seeking to draw on their multidisciplinary expertise, we hope that the insight emerging from our workshop will provide the essential, practical contextual background for this project and help to shape the parameters of our research inquiries.”

**Participants**

| **Project team (n=3)** | **Academics (n=9)** | **Healthcare sector (n=6)** |
| --- | --- | --- |
| Prof Karen Yeung, Project PI, Professor of Law, Ethics and Informatics, Birmingham Law School & School of Computer Science  Immaculate Motsi- Omoijade, Research Associate, Birmingham Law School  Dr Alex Kharlamov, Research Associate, Birmingham Law School | Prof Cristie Ford, Peter Allard School of Law, University of British Columbia, Vancouver  Dr David Galindo, Associate Professor in Computer Security at the University of Birmingham  Prof Andrew Howes, Professor, School of Computer Science, University of Birmingham  Prof Mireille Hildebrandt, Vrije Universiteit Brussels (VUB), Faculty of Law and Criminology & part-time Chair of Smart Environments, Data Protection and the Rule of Law at the Science Faculty, at the Institute for Computing and Information Sciences (iCIS) at Radboud University Nijmegen  Prof Mark Ryan, HP Inc Research Chair in Cyber Security, Director of Centre for Security and Privacy at the University of Birmingham  Dr Melek Somai, Assistant Prof Biomedical Informatics, Emory University  Dr Mark Taylor, Associate Professor in Health Law and Regulation, Melbourne Law School, University of Melbourne & Deputy Director, HeLEX@Melbourne.  Dr Chen Zhu, Lecturer in Intellectual Property Law, University of Birmingham | Dave Ebbit, Medicalchain, UK  Dr Indra Joshi, Clinical Lead for NHS England’s Empower the Person programme  William Nash, Dovetail Labs, Chief Operating Officer and co-founder  Dr Nathalie Pankova, Shivom, Chief Operating Officer  Dr Harpreet Sood (Associate Chief Clinical Information Officer (CCIO) at NHS England, Clinical practitioner, University College Hospital, London  Stewart Southey, Biohax International, Acting Chief Medical Officer |

**Workshop Proceedings**

*Day 1: structured discussion*

For each topic, a participant with relevant expertise and experience was invited to offer some introductory remarks, followed by interactive discussion.

Blockchain for healthcare

1. overview
2. medical records management
3. healthcare administration, supply chains and research management
4. technical, organisational, operational challenges
5. legal, ethical, and data governance challenges

*Day 2: Academics only*

1. Review of previous day’s discussion – exploration and reflection on key issues/domains of inquiry
2. Current state of development, testing and implementation of blockchain for healthcare applications
3. Significance, implications and prospects for utilising blockchain in healthcare
4. Challenges, risks and obstacles
5. Research methods and possible modes of inquiry

**Focus Group 2**

The second workshop took place on 28 November 2019 at The University of Birmingham.

**Academic-Stakeholder Workshop Materials:**

Workshop participants were provided with an information pack setting out the aim of the workshop discussion in the context of the Wellcome-funded research project, its research questions and overarching objectives. In particular, the welcome message from the project PI stated:

“The overarching aim of this project is to map the legal, ethical, technical and governance challenges pertaining to the regulation of healthcare through blockchain by identifying, mapping, and critically examining the implications for utilising Blockchain in this context.

This workshop will bring together a select group of key individuals involved in building blockchain applications for healthcare from the UK and USA, together with leading academics and from several disciplines including law, computer science, bioinformatics and medical ethics and clinicians, in order to share, reflect upon and critically discuss the provisional findings arising from our project. We then propose to refine and revise our findings in light of the workshop discussion, which should then enable us to complete academic papers for peer review publication, along with shorter more industry-focused publications for the healthcare sector.

We are indebted to you for kindly agreeing to join us for this event, recognising that your time is precious and limited. We hope that the insight and contacts that you gain from the workshop will be worthwhile

Discussion will take place under the Chatham House Rule (on the record but with no attribution) although participants are free to indicate if they would like particular comments to be made off-the-record.”

| **Project team (n=4)** | **Academics (n=8)** | **Healthcare sector (n=12)** |
| --- | --- | --- |
| Prof Karen Yeung, Project PI, Professor of Law, Ethics and Informatics, Birmingham Law School & School of Computer Science  Immaculate Motsi-Omoijade, Research Associate, Birmingham Law School  Dr Alex Kharlamov, Research Associate, Birmingham Law School  Katarzyna Ziolkowska, PhD candidate University of Warsaw and a visiting research student at the University of Birmingham | Prof Alastair Denniston, Consultant ophthalmologist University Hospitals Birmingham and Professor, University of Birmingham  Dr Alexandra Giannopoulou, lawyer and postdoctoral researcher, Blockchain and Society Policy Lab at the Institute for Information Law (IViR), University of Amsterdam  Dr David Galindo, Associate Professor in Computer Security at the University of Birmingham  Prof Andrew Howes, Professor, School of Computer Science, University of Birmingham  Dr Federica Lucivero. Senior Researcher in Ethics and Data, Ethox Centre and the Wellcome Centre for Ethics and Humanities, Big Data Institute, University of Oxford  Dr Maureen Mapp, Lecturer in Law, Birmingham Law School.  Dr Melek Somai, Assistant Prof Biomedical Informatics, Emory University  Dr Chen Zhu, Lecturer in Intellectual Property Law, University of Birmingham | Dr Abdullah Albeyatti, MedicalChain, CEO and co-founder  John Bass, Hashed Health, CEO and Founder  Helen Disney, Unblocked, CEO and Founder  Madeline Forster, Chatham House, international lawyer and research associate  Ashley Kerr, Mills & Reeve, Trainee Solicitor  Matt Lucas, IBM, Leader of IBM’s blockchain engagement team  Chris Miller, Chief Technology Officer, Guardtime Health  Jim Nasr, Certara. Vice-President of Technology & Innovation  Dr Nathalie Pankova, Metadvice, Chief Operating Officer  Dr Jonathan Passerat-Palmbach, ConsenSys Health, Director of Decentralised AI and Cryptography  Vincent Racine, Guardtime Health, solutions engineer  Dr Alex Szolnoki, Babylon Health, Comprehensive Health Record Team |

**Workshop Proceedings**

1. Introduction (by PI, Prof Karen Yeung)
2. Mapping the development of blockchain for healthcare: use cases and maturity (Immaculate Motsi-Omoijade)
3. Technological challenges and business opportunities: state of the art and future trajectories (Dr Alex Kharlamov, Jim Nasr responding)
4. Technological challenges and business opportunities: state of the art and future trajectories (Immaculate Motsi-Omoijade)
5. Critical Reflections (Prof Karen Yeung, Jim Bass responding)
